# Diurnal Temperature Variation and Plants Drive Latitudinal Patterns in Seasonal Dynamics of Soil Microbial Community

**DOI:** 10.3389/fmicb.2019.00674

**Published:** 2019-04-02

**Authors:** Ang Hu, Yanxia Nie, Guirui Yu, Conghai Han, Jinhong He, Nianpeng He, Shirong Liu, Jie Deng, Weijun Shen, Gengxin Zhang

**Affiliations:** ^1^Key Laboratory of Alpine Ecology and Biodiversity, Institute of Tibetan Plateau Research, Chinese Academy of Sciences, Beijing, China; ^2^College of Resources and Environment, Hunan Agricultural University, Changsha, China; ^3^Center for Ecology and Environmental Sciences, South China Botanical Garden, Chinese Academy of Sciences, Guangzhou, China; ^4^Key Laboratory of Ecosystem Network Observation and Modeling, Institute of Geographic Sciences and Natural Resources Research, Chinese Academy of Sciences, Beijing, China; ^5^Key Laboratory of Forest Ecology and Environment, Chinese Academy of Forestry, Beijing, China; ^6^School of Ecological and Environmental Sciences, East China Normal University, Shanghai, China

**Keywords:** seasonal microbial dynamics, temporal turnover, phylogenetic relatedness, ecological network, diurnal temperature variation, plants

## Abstract

Seasonality, an exogenous driver, motivates the biological and ecological temporal dynamics of animal and plant communities. Underexplored microbial temporal endogenous dynamics hinders the prediction of microbial response to climate change. To elucidate temporal dynamics of microbial communities, temporal turnover rates, phylogenetic relatedness, and species interactions were integrated to compare those of a series of forest ecosystems along latitudinal gradients. The seasonal turnover rhythm of microbial communities, estimated by the slope (*w* value) of similarity-time decay relationship, was spatially structured across the latitudinal gradient, which may be caused by a mixture of both diurnal temperature variation and seasonal patterns of plants. Statistical analyses revealed that diurnal temperature variation instead of average temperature imposed a positive and considerable effect alone and also jointly with plants. Due to higher diurnal temperature variation with more climatic niches, microbial communities might evolutionarily adapt into more dispersed phylogenetic assembly based on the standardized effect size of MNTD metric, and ecologically form higher community resistance and resiliency with stronger network interactions among species. Archaea and the bacterial groups of *Chloroflexi, Alphaproteobacteria*, and *Deltaproteobacteria* were sensitive to diurnal temperature variation with greater turnover rates at higher latitudes, indicating that greater diurnal temperature fluctuation imposes stronger selective pressure on thermal specialists, because bacteria and archaea, single-celled organisms, have extreme short generation period compared to animal and plant. Our findings thus illustrate that the dynamics of microbial community and species interactions are crucial to assess ecosystem stability to climate variations in an increased climatic variability era.

## Introduction

Climate change involves changes in the variability or average state of the atmosphere over time, which has profoundly disturbed and will continue to affect natural ecosystems and human life styles in the 21st century (Intergovernmental Panel on Climate Change [IPCC], 2014). Global temperature has experienced diurnally and seasonally asymmetric warming over the past decade with induced frequencies of extreme climatic events (Karl et al., 1991; Cohen et al., 2012). One of the grant challenges in ecology is to understand the variability and stability of biological communities in response to potential threats from climate change (Thomas et al., 2004; Fussmann et al., 2014). Resistance and resilience are usually estimated in relation to a community’s level of intrinsic variability (Shade et al., 2012). Although potential impacts of changes in average climatic conditions on the structure and temporal dynamics of biological communities have attracted attention worldwide (Woodward et al., 2010; Liang et al., 2015; Zhou et al., 2016), ecologists have only begun to understand the potential impacts of changes in climatic variability (Thompson et al., 2013; Vázquez et al., 2015; Chan et al., 2016; Wang and Soininen, 2017). It was stated that climatic variability could impact distribution patterns of macroorganisms due to their temperature tolerance and acclimation abilities. Given the fundamental role in microbial communities in biogeochemical cycling, their responses to climate change may be important determinants of ecosystem response to global change (Singh et al., 2010; Barberan et al., 2012). While microorganisms have short generation periods, compositions of soil microbial communities also varied seasonally (Habekost et al., 2008; Buckeridge et al., 2013; Smith et al., 2015). However, little is known whether the magnitude of climate variation selects for microorganisms, and how their community structure and seasonal dynamics vary with climate variation.

Although seasonal rhythm is fundamental to organisms and may be one of the strongest indicators of climate change, extensive study of seasonal variability on microbial communities has been reported only in aquatic ecosystems (Gilbert et al., 2012; Hatosy et al., 2013). Previous survey efforts to determine the factors affecting seasonal microbial community dynamics have mostly focused on the seasonal changes in environmental variables such as day length (Gilbert et al., 2012) or nutrient concentrations (Hatosy et al., 2013). Latitude, covering pronounced climatic gradients, is also one of the most intriguing factors possibly affecting the microbial community diversity and dynamics (Liang et al., 2015; Wang et al., 2016). Understanding the drivers of microbial community dynamics is important for predicting community response to climate change. However, seasonal dynamics of microbial communities and impacts of climate variation across latitudinal gradients are still understudied.

To study the impacts of climatic variability on soil microbial community structure and dynamics, we investigated seasonal variation of soil microbial communities and the underlying mechanisms in the face of global climate change. We selected five forest sites along a latitudinal gradient, spanning three different geoclimatic regions with considerable climatic variability, and compared the seasonal dynamics of microbial communities. Studies of community dynamics typically employ the time-decay relationship to characterize the temporal turnover rates. Such compositional turnover can be used for comparing microbial community resilience when challenged with different disturbances (Shade et al., 2012). Previous reports have shown that temporal turnover of biological communities may be driven by multiple factors, such as climate regions (Shurin et al., 2007), temporal (seasonal or annually) scales (Hatosy et al., 2013) and disturbances (Werner et al., 2007; Svensson et al., 2009). Furthermore, ecological networks among different species could help sustain ecosystem complexity, resilience and stability, and may affect ecosystem resistance to climate variation (Montoya et al., 2006; Tu et al., 2015). Therefore, we also analyzed the co-occurrence ecological network of microbial communities and the response to climate change.

## Materials and Methods

### Study Site and Sample Collection

Five forests with varied ecosystem types from subtropical to temperate forest along a latitudinal gradient of eastern China in long-term ecological research stations from Chinese Ecosystem Research Network (CERN) were selected (Supplementary Figure S1). These five forests are located in different climatic regions. Dinghu Forest (DHF; 23°10′30″ N, 112°33′12″ E) and Jinggang Forest (JGF; 26°40′01″ N, 114°23′34″ E), located in southern China, are southern subtropical forests and middle subtropical forests, respectively. Baotianman Forest (BTM; 33°33′34″ N, 111°42′29″ E), located in central China, is a transitional zone between warm temperate forests and north subtropical forests. Dongling Forest (DLF; 40°02′03″ N, 115°27′41″ E) and Changbai Forest (CBF; 41°59′17″ N, 127°56′19″ E), located in northern China, are warm temperate forests and typical temperate forests, respectively. Detailed information about sampling sites and these soil samples is listed in Supplementary Table S1. Soil samples were collected from these forests over three seasons for 2 years from 2013 to 2014, (1) spring (April–May, abbreviated “Sp”), (2) summer (July, abbreviated “Su”), and (3) autumn (October, abbreviated “A”). At each site, 10 soil samples from 10 separate plots (10 m × 10 m) were collected. Within each plot, 25 upper 10-cm soil cores were randomly taken using a soil auger (Φ 5 cm) to form one composite sample, which resulted in 60 soil samples for each forest site in the 2 years (300 soil samples in total). Visible gravel or plant detritus were removed prior to homogenizing each sample. Soil samples were sieved to 2 mm for physiochemical and molecular analyses.

### Climatic Variables

The climatic data were obtained from long-term monitoring dataset provided by CERN^1^. The climatic variables included were mean diurnal temperature range, mean temperature, temperature range, sum of precipitation and standard deviation of precipitation at the time scale of week, month, and season, respectively. Temperature measurements in our study reflect the air temperature. One-week mean temperature and sum of precipitation backward from the sampling date were defined as weekly temperature and precipitation for each season. Thirty-day mean temperature and sum of precipitation backward from the sampling date were defined as monthly temperature and precipitation for each season. Three-month mean temperature and sum of precipitation were defined as intra-seasonal temperature and precipitation for each season (i.e., Spring: March–May; Summer: June–August; Autumn: September–November). Weekly, monthly and intra-seasonal temperature range was computed as the difference between maximum and minimum temperature within a week, month, and season, respectively. Diurnal temperature range at the time scale of week, month, and season was computed as the difference between daily maximum and minimum temperature, then averaged within a week, month, and season, respectively. In addition, niche breadth was calculated as the variance of the standardized climatic variables (mean = 0; SD = 1) of the seasonal samples across the latitudinal gradient (Wang and Soininen, 2017). Niche breadth of temperature variation [DTR (intra-seasonal mean diurnal temperature range) and TR (intra-seasonal temperature range)] was calculated.

### Plant Variables

Gross primary productivity (GPP) refers to the total biomass fixed by the vegetation in an ecosystem during photosynthesis in a unit area within a unit time. GPP is associated with changes in plant phenology and climatic seasonality (Tomomichi et al., 2004; Lucy et al., 2014), and probably related to root and exudates turnover due to its allocation belowground (McCormack et al., 2014; Abramoff and Finzi, 2016). Therefore, we included GPP aspects to represent plant variables. GPP dataset was from the land processes distributed active archive center (LP DAAC) of the United States National Aeronautics and Space Administration (NASA) Earth Observing System (EOS)^2^. MODIStsp (v1.3.3) (Busetto and Ranghetti, 2016) was used to preprocess and extract time series data (from 2013 to 2014) at five forest sites. Monthly GPP averages were computed firstly, and intra-seasonal mean and standard deviation (SD) of GPP for each season (i.e., Spring: March–May; Summer: June–August; Autumn: September–November) were then computed. In addition, leaf area index (LAI, including tree, shrub and tree) and litterfall (including branch, leaf, fruit, and bark) aspects were also obtained to represent plant variables from long-term monitoring dataset provided by CERN (see footnote 1). The intra-seasonal mean and standard deviation (SD) of LAI and litterfall variables for each season were included in the following statistical analyses.

### Soil Physiochemical and Molecular Analyses

For each sample, 500 g of soil sieved to 2 mm was stored at 4°C for physiochemical analysis, and 50 g of soil was kept at -80°C for molecular analysis. Soil geochemical properties were measured according to the methods as described in He et al. (2017), including soil pH, WC (water content), TOC (total organic carbon), TON (total organic nitrogen), TP (total phosphorus), NH_4_^+^ (ammonium), NO_3_^-^ (nitrate), and DOC (dissolved organic carbon) (He et al., 2017). Soil molecular analyses included DNA extraction, PCR amplification and 16S rRNA gene sequencing. DNA was extracted from 0.5 g of frozen soil samples using FastDNA^®^ SPIN Kit for Soil (MP Biomedicals, Santa Ana, CA, United States) following the manufacturer’s instructions. DNA quality was assessed by a NanoDrop ND-2000c UV-Vis spectrophotometer (Thermo Fisher Scientific, Pittsburgh, PA, United States) and used in the downstream molecular analyses. The PCR amplification of the 16S rRNA gene hypervariable region V4 was performed with the primers 515F and 806R (515F: 5′-GTGCCAGCMGCCGCGGTAA-3′; 806R: 5′-GGACTACHVGGGTWTCTAAT-3′). The 5′-end of the reverse primers were fused to a sample barcode sequence. Template preparation was performed using Ion PGM^TM^ template OT2 400 kit (catalog No. 4479879, 4479880; Life Technologies, United States) according to the supplier’s instructions. Library of DNA fragments was done by ligating the adapters to the PCR products, and then clonally amplified onto the proprietary Ion Sphere^TM^ particles by emulsion PCR. The particles coated with template were then loaded onto the Ion chip. The chip was placed on the Ion Torrent Personal Genome Machine (PGM) system. The DNA sequences from each library were processed and filtered with PGM software to remove low-quality sequences. The preprocessing of the ion Torrent sequencing data and the downstream analysis were performed using Trimmomatic-0.33 (Bolger et al., 2014), Mothur (v. 1.36.0) (Schloss et al., 2009), QIIME (v. 1.8.0) (Caporaso et al., 2010b) and an in-house Galaxy software platforms (IEG sequence analysis pipeline^3^). The preprocessing of the ion Torrent sequencing data and the downstream analysis were as follows. All raw sequences were first converted to fastq format using samtools (Li et al., 2009). Quality control of the data was performed by filtering poor reads with a quality score cutoff of 20, removing the reads contained ambiguous bases or homopolymers greater than 8 bp in length and trimming the reads shorter than 150 bp and longer than 300 bp. Across all samples, a total of 6,331,592 high-quality sequences were obtained, which ranged from 7,116 to 60,422 sequences per sample with the average length of 227 bp. Reads that passed quality control were imported into Galaxy for the following analysis. In the Galaxy analysis pipeline, chimeras were removed using UCHIME (Edgar et al., 2011), and then chimera-free sequences were clustered to generate operational taxonomic units (OTUs) with a cutoff value of 97% sequence identity using UPARSE (Edgar, 2013). The OTU table that has singletons removed was used for the community analyses. Rarefaction curve was calculated (Supplementary Figure S2) and different communities were compared through equal amount of sampling size (subsampled to 7,000 sequences per sample). Taxonomic classification was performed in Galaxy with the RDP Classifier with a confidence threshold of 0.5 ( Wang et al., 2007). Representative OTU sequences from UPARSE were aligned using PyNAST (Caporaso et al., 2010a) with Greengenes database (DeSantis et al., 2006), and then the Newick formatted phylogenetic tree was built using FastTree (Price et al., 2010) for further phylogenetic analysis.

### Phylogenetic Community and Diversity Analyses

To determine whether different samples formed unique phylogenetically related clusters, principal coordinate analysis (PCoA) of the weighted Unifrac distance matrices was performed. UniFrac is a beta-diversity measure that uses phylogenetic information to compare microbial community composition among samples (Lozupone and Knight, 2005). A two-dimensional PCoA plot was created in QIIME and visualized by EMPeror (Vazquez-Baeza et al., 2013). Microbial community diversity index (Chao1) (Chao et al., 2009) was then calculated from the rarefied OTU profiles.

In order to measure the phylogenetic relatedness of microbial communities, the level of phylogenetic clustering of soil microbial communities in each forest ecosystem was tested. Mean nearest taxon distance (MNTD) of all species pairs occurring in a community based on the observed community dataset was calculated to estimate the mean phylogenetic relatedness between each OTU in a community and its nearest relative (Webb et al., 2002; Wang et al., 2013). The differences in the phylogenetic distances between the observed and randomly generated null communities were further computed, and then standardized using the standardized deviation of phylogenetic distances in 999 null communities (Webb, 2000). The obtained standardized effect size (ses.MNTD) can be used to test for phylogenetic clustering or overdispersion (Webb, 2000). Negative ses.MNTD values and low quantiles (*P* < 0.05) indicate that co-occurring species are more closely related than expected by chance (clustering), while positive values and high quantiles (*P >* 0.95) indicate less closely related species (overdispersion). These analyses were performed by using “picante” package (v.1.6-2) in R (v.3.0.1^4^) (Kembel et al., 2010).

### Co-occurrence Ecological Network Construction and Analysis

In order to understand how microbial communities assemble across the latitudinal gradient, co-occurrence ecological networks were constructed and analyzed using an open-accessible molecular ecological network analysis (MENA) pipeline^5^. Seasonal samples within each forest were combined for the analysis of co-occurrence network (networks were also constructed in each year individually within each forest). As previously applied, we focused on the core OTUs that were detected in more than 50% of the samples across each forest ecosystems (Deng et al., 2012). The same or very close threshold was applied to construct microbial co-occurrence networks, with the purpose of comparing different networks across a latitudinal gradient. This approach is remarkable in that the network is automatically defined and robust to noise, and the whole process and details are given in a previous MENA study (Deng et al., 2012). The network was visualized using Cytoscape 3.2.1 (Smoot et al., 2011).

Topological indexes for individual nodes in the network were calculated using the MENA pipeline. This feature set included node degree (the number of neighbors, also called connectivity), betweenness centrality (the number of shortest paths going through a node), stress centrality (the number of geodesic path that pass through a node) and clustering coefficient (the probability that the adjacent nodes of a node are connected, also called transitivity). The node betweenness centrality and stress centrality features were used to measure the centrality of each node in the network (Ma et al., 2016). Moreover, network-level topological features were also calculated for each network across latitudinal forest ecosystems. This feature set included node numbers (Nodes), edge numbers (Links), average clustering coefficient (avgCC), average path distance (GD), modularity (M), centralization of betweenness (CB) and centralization of stress (CS).

### Statistical Analyses

Adonis test for permutational multivariate analysis of variance based on weighted UniFrac distance was conducted to evaluate significant differences in community composition among seasons and between latitudinal forests. One-way ANOVA was used for testing the significance of latitudinal or seasonal differences in microbial data followed by a Tukey HSD or Games-Howell test using SPSS Statistics (20.0) Software (IBM). To test for differences in network topological features between latitudinal forests, multiple comparison of Kruskal–Wallis test was used. Multiple comparison analyses were performed by using the “agricolae” package in R. To compare the magnitude of variances of ses.MNTD values for phylogenetic groups of microbial communities between latitudinal forests, we performed an *F* test in R.

The time-decay relationship is an important indicator of the seasonal dynamics of microbial communities. To assess the temporal turnover rate of microbial community, linear regressions were used to examine the relationship between the temporal distance among samples and similarity in microbial composition. Temporal dynamics of the whole microbial community and different phylogenetic groups were explored at seasonal time scale (turnover rate was calculated in each year individually, and also calculated in 2 years combined). Archaea and major bacterial phyla were selected based on their relative abundances higher than 1%. *Proteobacteria* phylum was divided into different classes because of the high relative abundance and different ecological functions of these classes. The phylogeny-based weighted UniFrac distance was used as a metric of differences in community composition. Arrhenius (log–log) plot was used for modeling the species-time relationship in the form: log_10_(*S_s_*) = constant ± *w* log_10_(*T*), where *S_s_* is the pairwise similarity in community composition, *T* is the time interval and *w* is a measure of the rate of species turnover across time. Because our data consisted of pairwise comparisons and thus were not independent, bootstrapping (1,000 times) was used to test if the slope of the regression (*w* value) was significantly different from zero. A one-sample *t*-test between the original slope and a mean of bootstrapped slopes by random pairing of the original set (permuted 1,000 times) was conducted (Horner-Devine et al., 2004; Xiong et al., 2015). The significance comparison of *w* values among different estimations was also achieved by bootstrapping (1,000 times), followed by a pairwise *t*-test.

To estimate the contribution of climate, plant, soil, and spatial factors to microbial community structures and seasonal dynamics, multiple ordinary least squares (OLS) regression and the quantification of relative importance and variation partitioning analysis (VPA) were used. Spatial variables were obtained by principal coordinates of neighbor matrices (PCNM) which precisely represents the spatial relationship between the samples (Ramette and Tiedje, 2007). The first two spatial scales (PCNM1 and PCNM2) with positive values of Moran’I index were selected for multiple OLS regression and VPA analyses. Seasonal turnover rates and network topological features calculated in each year individually were used to increase data points for better modeling. Environmental variables were firstly selected for regression analyses. Strong correlated variables were dereplicated according to their correlation (i.e., one of the two variables was selected if the Pearson correlation is higher than 0.8). In multiple OLS regression analysis, all of the microbial features and environmental variables were standardized at a mean of 0 and SD of 1. Akaike’s information criterion was used to identify the best model. Multiple OLS regression analyses were performed by using the “MASS” package in R. Then, each regressor’s contribution to a multiple OLS regression model was quantified. The R “relaimpo” package provides measures of relative importance with the lmg method for each of the predictors in the model. For VPA, climate, plant, soil and spatial variables were forward selected by the “forward.sel” function with R “packfor” package, respectively. VPA was carried out by using the R “vegan” package. The Pearson correlation (two-tailed) was used to identify the relationships between microbial features and environmental variables in R.

## Results

### Seasonal Changes of Environmental Factors Across the Latitudinal Gradient

Climatic conditions at the time scale of season were highly varied along the latitudinal gradient (Supplementary Figure S3): intra-seasonal mean diurnal temperature range (DTR) and intra-seasonal temperature range (TR) increased significantly with increasing latitudes, whereas intra-seasonal mean temperature (AT) and intra-seasonal sum of precipitation (Precip) decreased significantly with increasing latitudes; TR and AT showed periodic patterns with a trough and peak in summer in all forests, respectively. For plant variable, GPP was used. Monthly GPP averages at northern latitudes had a stronger seasonal pattern, while those at southern latitudes maintained higher levels across all seasons though somewhat reduced during winter time (Supplementary Figure S4). GPP had higher correlations with climatic factors (temperature and precipitation) at northern (*P* < 0.01) than southern latitudes (*P* > 0.05; Supplementary Figure S4). For soil properties, soil pH value was lower at southern than northern forests, and nutrient levels, such as TOC, TN, DOC and available nitrogen including NH_4_^+^ and NO_3_^-^ were generally higher in northern than southern forests (data not shown).

### Microbial Community Structure Changes Across the Latitudinal Gradient

**FIGURE 1:**
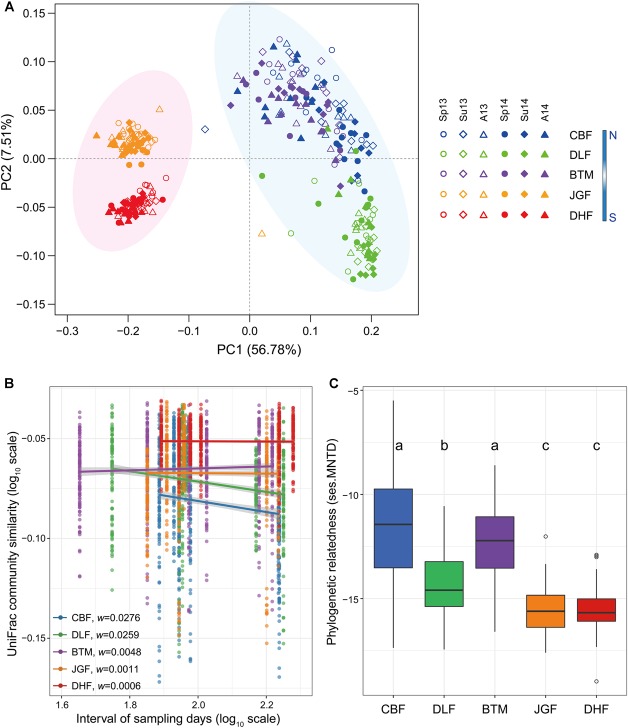
Seasonal dynamics of microbial communities along the latitudinal forest ecosystems. Principal coordinate analysis (PCoA) plot of phylogenetic microbial community using the weighted UniFrac distance metric **(A)**. Microbial temporal turnover **(B)** and phylogenetic relatedness (ses.MNTD, **(C)**. The turnover rate, *w* (the regression slope), was estimated using a linear regression (log–log space approach) fit between the pairwise average similarity values and intervals of sampling time at seasonal temporal scales across latitudinal forest ecosystems. The slopes of all lines were significantly different from zero and significantly different for pairwise comparison. Solid lines indicate the predicted relationships are significant (*P* < 0.05) based on linear regression estimated using ordinary least squares. Linear relationships at different latitudinal forests are indicated by color, and the shaded region represents the 95% confidence limits on the regression estimates. The standardized effect sizes of MNTD (ses.MNTD) values were all significantly negative (*P* = 0.001). Pairwise comparison was performed between latitudinal samples. Different letters (a, b, c) indicate a significant difference (*P* < 0.05) by ANOVA analysis. Forests along a latitudinal gradient from north to south include Changbai Forest (CBF), Dongling Forest (DLF), Baotianman Forest (BTM), Jinggang Forest (JGF), and Dinghu Forest (DHF). N, northern sites; S, southern sites. Samples were coded by sampling season and year. Sp, spring; Su, summer; A, autumn. The number 13 and 14 represents Year 2013 and 2014, respectively.

We found clear differentiation in microbial community composition between southern (DHF and JGF) and central (BTM) and northern (DLF and CBF) latitudinal forests (adonis test for permutational multivariate analysis of variance, *F* = 98.5, *P* = 0.001; Figure 1A). Forest latitude, compared to seasonal variation, was the major driver in microbial community differentiations, with the former accounting for 90.81% and the latter for 0.27% of total variation (Supplementary Table S2). In addition, alpha diversity index (Chao1) was significantly lower at southern latitudes (DHF and JGF) than that at central (BTM) and northern (DLF and CBF) forests (ANOVA, *P* < 0.05; Supplementary Figure S5a), and with the greatest seasonal fluctuation in DLF (*F* = 17.68, *P* < 0.001; Supplementary Figure S5b).

### Temporal Turnover Rates of Microbial Community Across the Latitudinal Gradient

The time-decay relationship is an important indicator of the seasonal dynamics of microbial communities. To evaluate the differences in microbial temporal turnover rates across latitudes, we estimated the slopes of microbial time-decay relationship. Significant time-decay relationships (*P* < 0.001) were observed for microbial communities at all latitudes, but faster seasonal turnover rates were found at northern latitudes (Figure 1B). Compared with central latitude (BTM, *w* = 0.0048), the rates of temporal turnover increased significantly toward higher latitudes (*w* = 0.0259 in DLF and 0.0276 in CBF, *P* < 0.001), whereas they declined significantly toward lower latitudes (*w* = 0.0011 in JGF and 0.0006 in DHF, *P* < 0.001) (Figure 1B). Similarly, the trend of declining temporal turnover rates with decreasing latitudes was also observed in each year individually (Supplementary Figure S6). We also observed considerable variations of turnover rates among different phylogenetic groups (Supplementary Table S3). Several phylogenetic groups exhibited distinct temporal turnover rates at northern versus southern latitudes. Specifically, Archaea and the bacterial groups of *Chloroflexi, Alphaproteobacteria*, and *Deltaproteobacteria* had greater turnover rates at northern than southern latitudes, whereas *Verrucomicrobia* had greater turnover rates at southern latitudes; the turnover rates of *Bacteroidetes, Betaproteobacteria*, and *Gammaproteobacteria* were weakly associated with latitudes (Supplementary Table S3).

### Phylogenetic Relatedness of Microbial Communities Across the Latitudinal Gradient

In order to measure the phylogenetic relatedness of microbial communities, we tested the level of phylogenetic clustering of soil microbial communities in each forest ecosystem. All the standardized effect sizes of mean nearest taxon distance (ses.MNTD) were all significantly negative (*P* = 0.001, Figure 1C), which indicated that microbial communities had a tendency to be more phylogenetically clustered than expected by chance. Moreover, this clustered community assembly patterns tended to weaken significantly (ANOVA, *P* < 0.05) at central and northern (BTM, DLF, and CBF) than southern (JGF and DHF) latitudinal forests (Figure 1C). We also compared variances of ses.MNTD between latitudinal forests among different phylogenetic groups (Supplementary Figure S7). Several phylogenetic groups, namely, Archaea, *Actinobacteria, Chloroflexi, Firmicutes*, and *Deltaproteobacteria*, exhibited considerable variances of ses.MNTD at northern versus southern latitudes.

### The Co-occurrence Networks of Microbial Communities Across the Latitudinal Gradient

**FIGURE 2:**
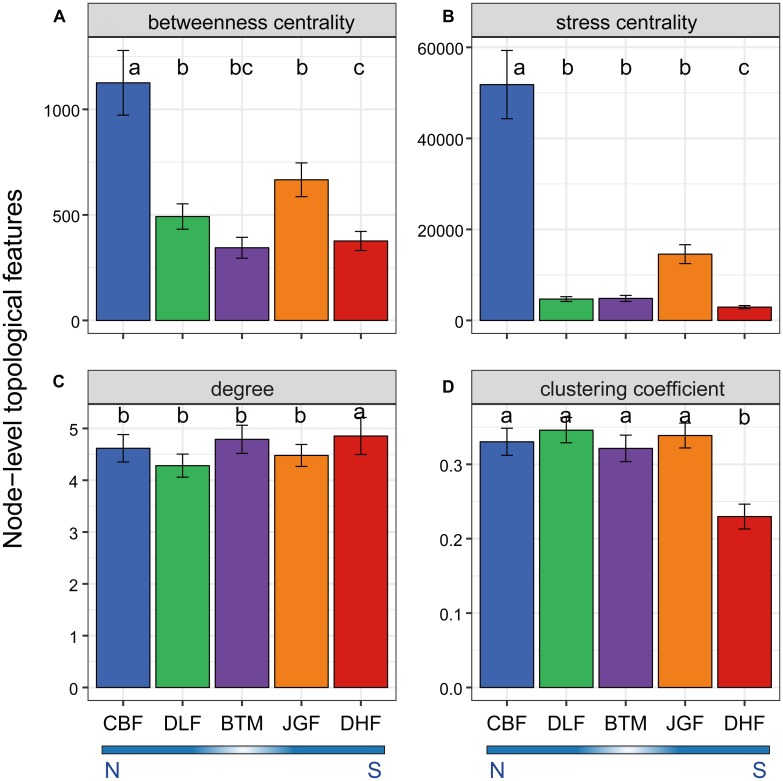
Node-level topological features in the network for the microbial community across latitudinal forest ecosystems. The topological features include betweenness centrality **(A)**, stress centrality **(B)**, degree **(C)**, and clustering coefficient **(D)**. Data are means ± SE, *n*, node numbers. Pairwise comparison was performed between latitudinal samples. Different letters (a, b, c) indicate a significant difference (*P* < 0.05) by Kruskal–Wallis test. N, northern sites; S, southern sites.

In order to understand how microbial communities assemble across the latitudinal gradient, co-occurrence networks were constructed for microbial communities at each forest site (Supplementary Figure S8). We then examined whether microbial OTUs associated with a specific latitudinal region exhibited unique node-level topological features. Firstly, we examined node betweenness centrality and stress centrality which are proxies for the location of this node in relation to other nodes, and observed significantly higher betweenness and stress centrality values (*P* < 0.05, Kruskal–Wallis test) at northern than southern latitudes (Figure 2A,B). High centrality values indicate a core location of this node in the network, and nodes with high centrality values are likely to have high influence on other interactions in the community. This suggests that microbial communities from the northern forest ecosystems were more often located in core, central positions within the network and had a higher interaction influence than those from the southern forest ecosystems. Secondly, we showed that node degree was slightly higher in one southern forest (DHF; *P* < 0.05, Kruskal–Wallis test; Figure 2C), but clustering coefficient was significantly lower at DHF (*P* < 0.001, Kruskal–Wallis test, Figure 2D). Furthermore, most of network-level topological features, such as average clustering coefficient (avgCC), modularity (M) and centralization of betweenness (CB), visually appeared relatively higher at northern than southern forest ecosystems (Supplementary Figure S9). These indicate that the network exhibited greater complexity and higher connectivity in the northern forests (CBF and DLF). In contrast, average path distance (GD) of the network was lower at southern latitudes compared to that at northern latitudes (Supplementary Figure S9 and Supplementary Table S4), which suggests a closer relationship in the southern forests (JGF and DHF). In addition, a few more node numbers, link numbers as well as negative links were observed at southern latitudes (JGF and DHF; Supplementary Figure S9 and Supplementary Table S4).

### Linking Microbial Communities and Seasonal Dynamics to Environmental Factors

**FIGURE 3:**
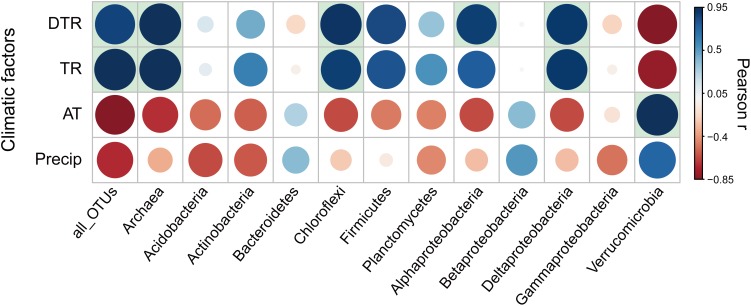
Pearson correlation coefficients (*r*) between temporal turnover rate for each phylogenetic group of microbial communities and climatic factors. The significant (*P* < 0.05) correlation coefficients were indicated by filling the background of the grid with light blue. DTR, intra-seasonal mean diurnal temperature range; TR, intra-seasonal temperature range; AT, intra-seasonal mean temperature; Precip, intra-seasonal sum of precipitation.

For all taxonomic groups, microbial temporal turnover rate had a significantly (*P* < 0.05) positive relationship with temperature variation [i.e., intra-seasonal mean diurnal temperature range (DTR) and intra-seasonal temperature range (TR)] (Figure 3). Niche breadth of temperature variation is positively associated with seasonal turnover rates of microbial communities (Supplementary Figure S10). In addition, greater seasonal dynamics of microbial communities per unit variation of diurnal temperature were observed at northern latitudes (Supplementary Figure S11). For each phylogenetic group of microbial communities, the temporal turnover rate showed differences in sensitivity to temperature variation (Figure 3). Specifically, the turnover rates of Archaea and the bacterial groups of *Chloroflexi, Alphaproteobacteria*, and *Deltaproteobacteria* had significantly (*P* < 0.05) positive correlation with temperature variation (i.e., DTR and TR); the turnover rates of *Bacteroidetes, Betaproteobacteria*, and *Gammaproteobacteria* were weakly associated with temperature variation (Figure 3). Some genera in the phylogenetic group of *Alphaproteobacteria* that was sensitive to temperature variation had significant correlation with temperature variation (Supplementary Table S5). Specifically, the turnover rates of *Rhizobium* had significantly (*P* < 0.05) positive correlation with DTR while the rate of *Bradyrhizobium* and *Rhizomicrobium* had significantly (*P* < 0.05) negative correlation with temperature variation (i.e., DTR and TR).

**FIGURE 4:**
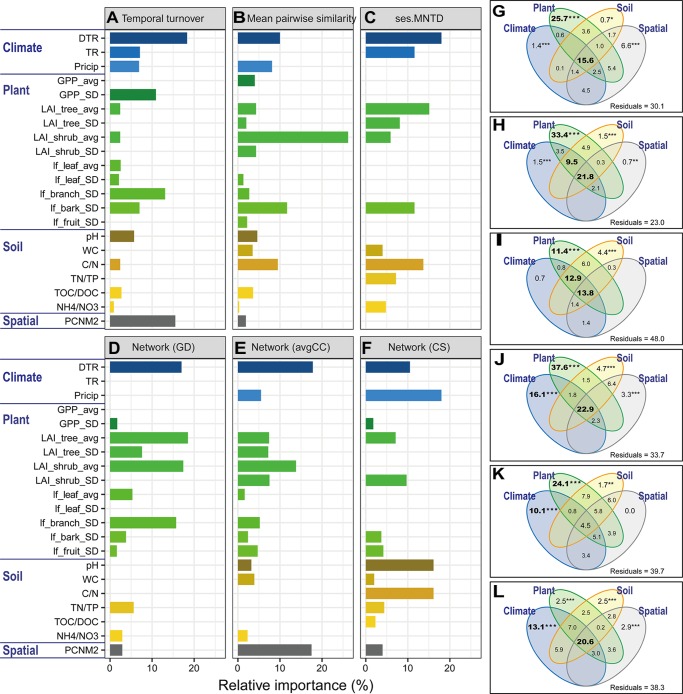
Relative importance of environmental factors related to the microbial features. The relative importance was identified with a linear model based on multiple ordinary least squares (OLS) regression **(A–F)** and variation partition analysis (VPA, **(G–L)**. Microbial features include temporal turnover rate (*w* values, **(A,G)**, mean pairwise UniFrac similarity **(B,H)**, phylogenetic relatedness (ses.MNTD, **(C,I)** and network-level topological features (GD, avgCC, and CS; **(D–F,J–L)**. The values of the relative importance (%) of each variable for each microbial metric in the model are shown as bar plots. The best models were identified using Akaike’s information criterion. All of the environmental variables were standardized (mean = 0; *SD* = 1). The explanatory environmental variables were summarized based on the results of multiple OLS regression (see Supplementary Table S6 for details). GD, average path distance; avgCC, average clustering coefficient; CS, centralization of stress. Environmental factors are divided into groups of climate (DTR, intra-seasonal mean diurnal temperature range; TR, intra-seasonal temperature range; Precip, intra-seasonal sum of precipitation), plant (GPP_avg, intra-seasonal mean of GPP; GPP_SD, intra-seasonal standard deviation of GPP; LAI_tree_avg, intra-seasonal mean of tree leaf area index; LAI_tree_SD, intra-seasonal standard deviation of tree leaf area index; LAI_shrub_avg, intra-seasonal mean of shrub leaf area index; LAI_shrub_SD, intra-seasonal standard deviation of shrub leaf area index; lf_leaf_avg, intra-seasonal mean of leaf litterfall; lf_leaf_SD, intra-seasonal standard deviation of leaf litterfall; lf_branch_SD, intra-seasonal standard deviation of branch litterfall; lf_bark_SD, intra-seasonal standard deviation of bark litterfall; lf_fruit_SD, intra-seasonal standard deviation of fruit litterfall), soil (pH, soil pH; WC, water content; C/N, the ratio of total organic carbon and nitrogen; TN/TP, the ratio of total nitrogen and phosphorus; TOC/DOC, the ratio of total organic carbon and dissolved organic carbon; NH_4_^+^/NO_3_^-^, the ratio of ammonium and nitrate), and spatial (PCNM2) variables. Asterisks represent significance level: ^∗∗∗^*P* < 0.001, ^∗∗^*P* < 0.01, ^∗^*P* < 0.05.

Microbial community structures and seasonal variations were significantly (*P* < 0.05) correlated with spatial distance, climate, plant and soil variables (Supplementary Table S6). We further used multiple OLS regression and quantification of relative importance to estimate the contribution of individual environmental variables to microbial features (Figure 4A–F and Supplementary Figures S12a–d). DTR contributed the largest importance for microbial temporal turnover rate (18.3%), phylogenetic relatedness (ses.MNTD, 18.0%) and network-level topological features such as avgCC (17.8%). Precipitation (Precip) contributed the largest importance for network-level topological features such as CS (18.0%). DTR was an important variable for explaining mean pairwise UniFrac similarity (10.0%), first axis of PCoA scores (15.1%), gamma diversity (11.2%) and network topological features such as GD (17.0%), CS (10.5%), and CB (15.7%). TR was an important variable for explaining phylogenetic relatedness (ses.MNTD, 11.6%), alpha diversity (10.8%) and CB (10.1%). Moreover, plant variables (GPP, LAI of tree and shrub, litterfall of branch and bark) and their seasonal variation were important variables for explaining many microbial features. It is worth mentioning that spatial distance (PCNM variable) also contributed to the variation of some microbial features, such as temporal turnover rate (15.5%), avgCC (17.5%), alpha diversity (26.4%) and gamma diversity (15.3%). Furthermore, VPA was subsequently performed to dissect the relative contributions of spatial, climate, plant and soil variables to microbial features (Figure 4G–L and Supplementary Figures S12e–h). The interactions of those four groups of variables accounted for a large amount of the total variation (14–53%) of almost all of microbial features. Climate alone accounted for 10–16% of the total variation of microbial network topological features such as GD, avgCC, CS, and CB. Plant alone accounted for 11–35% of the total variation of microbial temporal turnover, phylogenetic relatedness and network topological features. The interactions between climate and plant variables had large effects (9.5–13%) on the total variation of mean pairwise similarity and phylogenetic relatedness of microbial communities. Here, however, spatial variables alone explained slight variations of all of microbial features.

## Discussion

Dynamics properties of an ecological system is sometimes assessed by the level of variability in community compositions over time, as represented by temporal turnover rates (Gonze et al., 2018). The temporal turnover of microbial communities has been linked to a variety of factors such as latitude. We observed higher seasonal turnover rates of microbial communities at northern than southern latitudes. Temporal turnover has been well documented in macroorganisms, but reports on microbial temporal turnover remain limited (Shade et al., 2013). Latitude is one of the most intriguing factors that affect the rate of temporal turnover, yet there was not a consistent trend in temporal turnover with latitude for macroorganisms according to previous studies (Korhonen et al., 2010). For example, slower temporal turnover was observed at high latitudes in zooplankton communities (Shurin et al., 2007), whereas faster turnover was found at high latitudes in British birds (Evans et al., 2008). Temporal scale was also considered as one of the most significant factors that affect the rate of temporal turnover and previous research demonstrated that turnover in aquatic species (larger organisms or eukaryotes) was faster at lower latitude at intra-annual time scale, but the pattern was reversed at interannual time scale, where turnover was faster at high latitudes (Korhonen et al., 2010). Yet there was a lack of studies on latitudinal patterns of temporal turnover at different timescales for microbial communities (bacteria and archaea). Thus, for the first time, we have highlighted significant differences in latitudinal patterns of seasonal turnover rates for soil microbial communities.

To better understand how microbial communities and seasonal dynamics were spatially structured, we quantified the relative effects of spatial, climate, plant and soil factors that best explain the microbial variations. Microbial features could relate noticeably to significant variation in pure climate or plant effect, and to joint effect of the two groups of variables. It is worth noting that a pure spatial effect was not expected to contribute to the microbial variation, but rather through the shared effect with environmental factors (i.e., climate, plant and soil factors). We suggest that environmental factors were also spatially structured, thus leading to similar spatial patterns in microbial community structures and seasonal dynamics across latitudinal gradient.

We found that temperature variation rather than mean conditions imposed stronger influence on seasonal fluctuations of microbial communities across the latitudinal gradient. The statistical analyses showed that DTR and TR were important variables for explaining microbial seasonal dynamics. Many studies previously discussed the effect of diurnal (Ali and Subhasis, 2015) and seasonal temperature variation (Chang et al., 2011a,b) on microbial community and activity. They also found that temperature variations could induce a change in soil microbial community structures and enhance microbial activities, compared to a constant average temperature mode. High latitudes are usually characterized by strong seasonal acuity within a short seasonal period (e.g., larger DTR and TR), which is concurrent with seasonal turnover rhythm of microbial communities (Figure 3). Consistently, a recent study documented that greater long-term (e.g., seasonal) climate variation was related to narrower community geographical range of thermal specialists, as found for aquatic species (macroinvertebrates, diatoms, and bacteria) (Wang and Soininen, 2017). This finding is not supportive of a temperature mechanism: organisms that experience greater temperature variation and thus have broader physiological thermal tolerances, tend also to be widely distributed as a consequence (Janzen, 1967; Chan et al., 2016). Compared with recent studies by Chan et al. (2016) and Wang and Soininen (2017), we used shorter-term climate variation (DTR and TR within a season rather than among seasons) due to shorter generation span of soil microbes which are ectothermic and thus perhaps more sensitive to the variation in ambient temperature especially at shorter timescales. Our observations showed that seasonal turnover rates for most taxonomic groups, especially Archaea and the bacterial groups of *Chloroflexi, Alphaproteobacteria*, and *Deltaproteobacteria*, positively correlated with temperature variation (DTR and TR) (Figure 3), indicating they were more sensitive to high temperature variation in high latitude areas. Coincidentally, microbial species within these four groups tend to be more phylogenetically dispersed at northern latitudes based on the variability in ses.MNTD values (Supplementary Figure S7). Thus, greater temperature variation might have imposed stronger selective pressure on thermal specialists, because they depend on narrow environmental range and are more susceptible to temperature variation (Vázquez et al., 2015), which is an explanation for the positive effect of temperature variation (DTR and TR) on microbial seasonal dynamics.

Seasonal changes of plants might be one of important explanations for microbial seasonal dynamics in response to the increased temperature variation. Plants, as primary producers, transport a large proportion of fixed photosynthetic carbon to the soil environment, and this partitioning of nutrients may directly influence microbial associations (Kuzyakov and Domanski, 2000). Previous studies found that plant is an important driver of seasonal microbial dynamics through belowground C allocation (Kaiser et al., 2011). Plant phenology, the timing of biological events, is intimately tied to the diurnal and seasonal variations in climate (McClung, 2006; Visser et al., 2010; Helm et al., 2013; Thackeray et al., 2016). Diurnal temperature variation (DTR), associated with solar radiation and photoperiod, was found to regulate plant functions, including central carbon metabolism, stomatal opening, and the timing component of photoperiodism, which regulate seasonal reproduction such as flowering and the transition from vegetative to reproductive growth (Michael et al., 2003). For one thing, higher diurnal temperature variation would promote net assimilation, i.e., increase photosynthesis during the light period and decrease respiration during the dark period (Yang et al., 2016); for another, increased day length during spring and summer (in the northern hemisphere) would accelerate plant reproduction phase, i.e., compress the length of growth cycle (Michael et al., 2003). Evergreen trees at southern latitudes continue photosynthesizing (though somewhat reduced) during winter time, while deciduous trees at central and northern latitudes have a stronger seasonal pattern in photosynthesis and its allocation belowground due to much shorter phenological growing periods (data not shown). At higher latitudes, stronger seasonal belowground allocation of photosynthetic carbon might be related to faster litterfall, root and exudates turnover, probably leading to high variance in nutrient availability among seasons (Burke and Raynal, 1994; McCormack et al., 2014), resulting in a higher seasonal dynamics of microbial community compositions consequently. Our results showed that plant alone and the interactions among climate, plant and soil variables were considerable explanations for faster temporal turnover at higher latitudes. These further supported the idea that microbial variation may be generated by an indirect effect of temperature variation through seasonal patterns of plants besides a direct effect.

The response of microbial communities directly and indirectly to increased temperature variation could have implications on the following aspects.

First, temperature variation is likely associated with shifts in ecological niches (the particular set of resources and environmental conditions that an individual species exploits) (Prosser et al., 2007). Niche breadth of temperature variation is consistent with microbial seasonal dynamics (Supplementary Figure S10). Phylogenetic niche conservatism hypothesis holds that close relatives occupy similar niches, whereas distant relatives are more dissimilar (Wiens et al., 2010). Microbial species tend to be more phylogenetically clustered at southern latitudes, compared with those at northern latitudes based on ses.MNTD values (Figure 1C). These suggest more niches in northern forests due to more variable temperature and faster turnover of plant (i.e., litterfall, root and exudates) conditions, which consequently contribute to more highly dynamic microbial communities at higher latitudes.

Second, microbial network structure and interaction has been proposed as a determinant of community dynamics (Faust et al., 2018). Microbial communities from the northern forest ecosystems were more complex and had a greater interaction influence than those from the southern forest ecosystems based on node centrality values and network-level topological features. Relationships (i.e., mutualism or cooperation) between different microbial taxa with the seasons could be strengthened by providing faster resources turnover and more variable environmental conditions at northern latitudes. The interdependencies on resources and environmental conditions likely promoted beneficial interactions among community members, and microbes could thus extend their fundamental niches to adapt to dynamic environments (Hassani et al., 2018). Complex interaction networks may produce greater stability which could dampen the rapid spread of disturbance in a community (Olesen et al., 2007) and provide a buffer against environmental variation (Konopka et al., 2014). Microbial network interactions have been reported to be enhanced by environmental fluctuations (Xiong et al., 2015). These imply that intensified interactions of species co-occurrence might contribute to community resistance to greater temperature variation in the northern regions.

Third, in addition to community resistance, the resiliency may also influence the response of microbial communities to the increased climatic variation. Higher latitudes had greater fluctuations of microbial communities per unit variation of diurnal temperature (Supplementary Figure S11). Compositional turnover determined using similarity-decay approach can be used for comparing microbial community resilience when challenged with different disturbances (Shade et al., 2012). We infer that microbial communities at higher latitudes might have greater resiliency in response to the increased climatic variation. In addition, our results showed higher microbial diversity (Chao1; Supplementary Figure S5) and less phylogenetically clustered microbial species (Figure 1C) at higher latitudes. Ecosystem resilience is normally tied to the biodiversity; that is, maintaining biodiversity is a key to maintaining ecosystem resilience and avoiding thresholds at which the ecosystem loses its capacity to recover (Thompson, 2011; Oliver et al., 2015).

## Conclusion

The present study demonstrated that the seasonal turnover rhythm of microbial communities was spatially structured across the latitudinal gradient, which may be caused by a mixture of both diurnal temperature variation and seasonal patterns of plants. For the first time, to the best of our knowledge, we showed that climatic variation was more important than average environment drivers for determining the response of soil microbial community dynamics to climate change. The results might be supportive of an explanation that greater temperature variation imposes stronger selective pressure on thermal specialists; for instance, Archaea and the bacterial groups of *Chloroflexi, Alphaproteobacteria*, and *Deltaproteobacteria* are more sensitive to temperature variation. Plant, tied to the seasonal variations in climate, was also a considerable explanation for microbial seasonal dynamics in response to the increased temperature variation probably through seasonal belowground photosynthetic carbon flow. Furthermore, less clustering level of phylogenetic structure of microbial communities and stronger interaction intensity of species co-occurrence were potential explanations for highly dynamic microbial communities with greater resistance and resiliency in response to climatic variation at high latitudes. Our findings provide evolutionarily and ecologically mechanistic explanations on the community level, and have important implications for assessing ecosystem stability to climatic variation caused by global warming.

## Data Availability

The raw sequencing data for the 16S rRNA genes are publicly available in the NCBI Short Read Archive under accession no. PRJNA523913.

## Author Contributions

All authors contributed the intellectual input and assistance to this study and manuscript preparation. GY, WS, and GZ developed the original concepts. YN, CH, JH, NH, and SL contributed the reagents, collected the data, and performed the data analysis. AH and YN performed the statistical analysis and wrote the manuscript with help from JD and GZ. All authors reviewed results and commented on the manuscript.

## Conflict of Interest Statement

The authors declare that the research was conducted in the absence of any commercial or financial relationships that could be construed as a potential conflict of interest.

## Footnotes

1 http://cerndis1.cern.ac.cn

2 https://lpdaac.usgs.gov/dataset_discovery/modis/modis_products_table

3 http://zhoulab5.rccc.ou.edu

4 http://r-forge.r-project.org

5 http://ieg2.ou.edu/MENA
