# Supplementary material for: Diurnal Temperature Variation and Plants Drive Latitudinal Patterns in Seasonal Dynamics of Soil Microbial Community
Source: Front Microbiol. 2019 Apr 2;10:674. doi: 10.3389/fmicb.2019.00674 (PMC6454054; doi:10.3389/fmicb.2019.00674)
Supplement: Supplementary file 1 [file Data_Sheet_1.pdf]

Supplemental Information

Diurnal temperature variation and plants drive latitudinal patterns in seasonal dynamics of soil microbial community

Short running title: latitudinal patterns of microbial dynamics

Ang Hu [1,2,†], Yanxia Nie [3,†], Guirui Yu [4], Conghai Han [1], Jinhong He [3], Nianpeng He [4], Shirong Liu [5], Jie Deng [6], Weijun Shen [3], Gengxin Zhang [1,*]

[†] Contributed equally, * Corresponding Author

Contents of supplemental information

- **Supplementary Figures and Tables**
    - Figure S1: A view of forest sampling sites along the latitudinal gradient in Eastern China.
    - Figure S2: Rarefaction curve.
    - Figure S3: Latitudinal and seasonal changes of climatic factors across latitudinal forest ecosystems.
    - Figure S4: Gross primary productivity (GPP) and the relationship with climatic factors.
    - Figure S5: Latitudinal and seasonal changes of OTU richness (Chao1) across latitudinal forest ecosystems.
    - Figure S6: Time-decay for similarity relationship for the microbial community across latitudinal forest ecosystems at Year 2013 and 2014.
    - Figure S7. The phylogenetic relatedness for the phylogenetic groups of microbial communities across latitudinal forest ecosystems.
    - Figure S8: The network graph for the microbial community across latitudinal forest ecosystems.
    - Figure S9: Network-level topological features for the microbial community across latitudinal forest ecosystems.
    - Figure S10: Niche breadth of temperature variation along a latitudinal gradient and the relationship with temporal turnover rates of microbial communities.
    - Figure S11: The relationships between microbial community dynamics and temperature variation (DTR).
    - Figure S12: The relative importance of environmental factors related to the microbial features, identified with a linear model based on multiple ordinary least squares (OLS) regression and variation partition analysis (VPA).
    - Table S1: Summary of main characteristics of sampling sites along the latitudinal gradient in Eastern China.
    - Table S2: Quantitative effects of latitudes and seasons on the variation in the microbial community with adonis based on the weighted UniFrac distance.
    - Table S3: Temporal turnover rates of microbial communities among different phylogenetic groups.
    - Table S4: Topological properties of the microbial communities' phylogenetic molecular ecological networks (MENs) across latitudinal forest ecosystems and their associated random MENs.
    - Table S5: Temporal turnover rates of microbial communities among different phylogenetic groups at the genus level, and their relationships with climatic factors.
    - Table S6: Relationships between microbial features and potential explanatory variables that were modeled using multiple ordinary least squares (OLS) regression.

Supplementary Figures

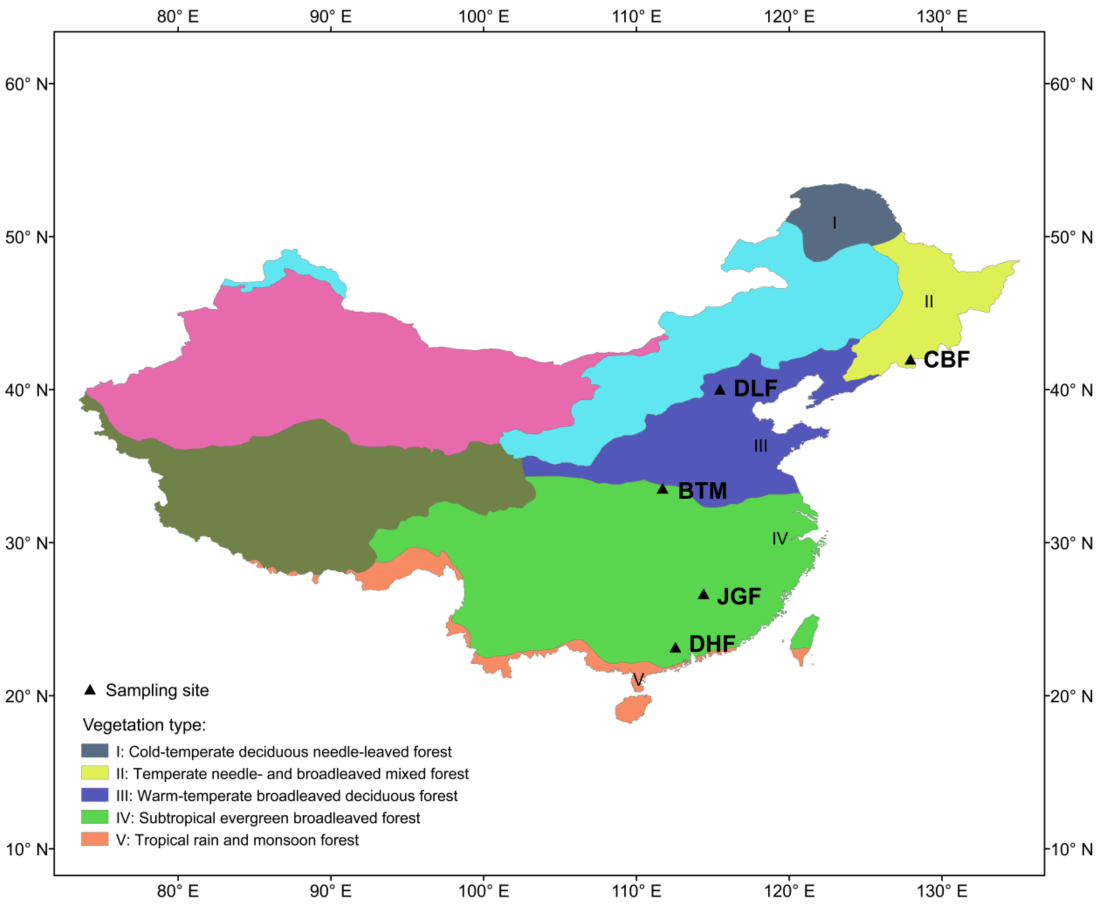

Figure S1. A view of forest sampling sites along the latitudinal gradient in Eastern China: CBF, Changbai Forest; DLF, Dongling Forest; BTM, Baotianman Forest; JGF, Jinggang Forest; and DHF, Dinghu Forest.

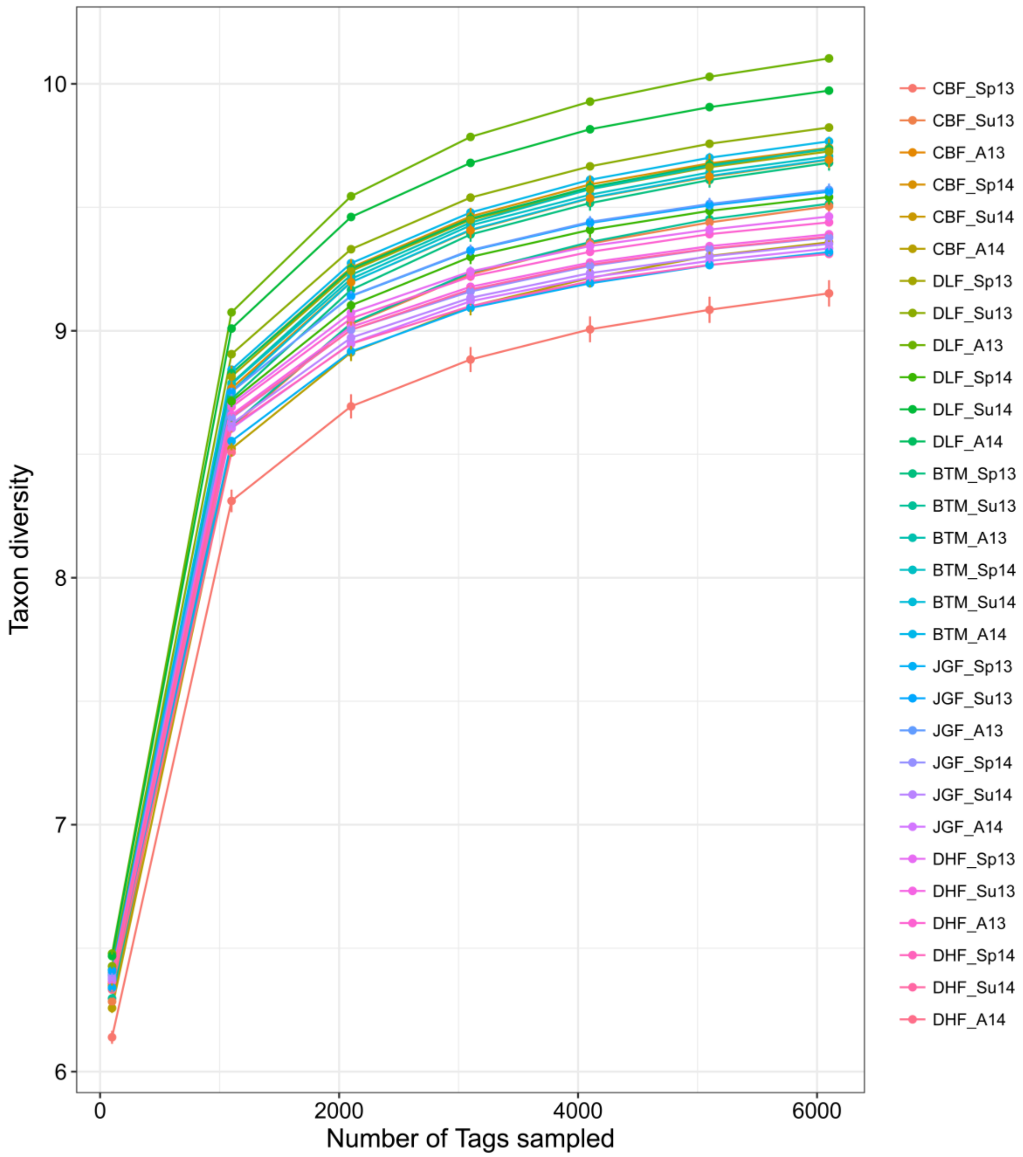

Figure S2. Rarefaction curve showing the relationship between the increase of microbial diversity and the number of randomly sampled sequences. Taxon diversity predicted by Shannon index.

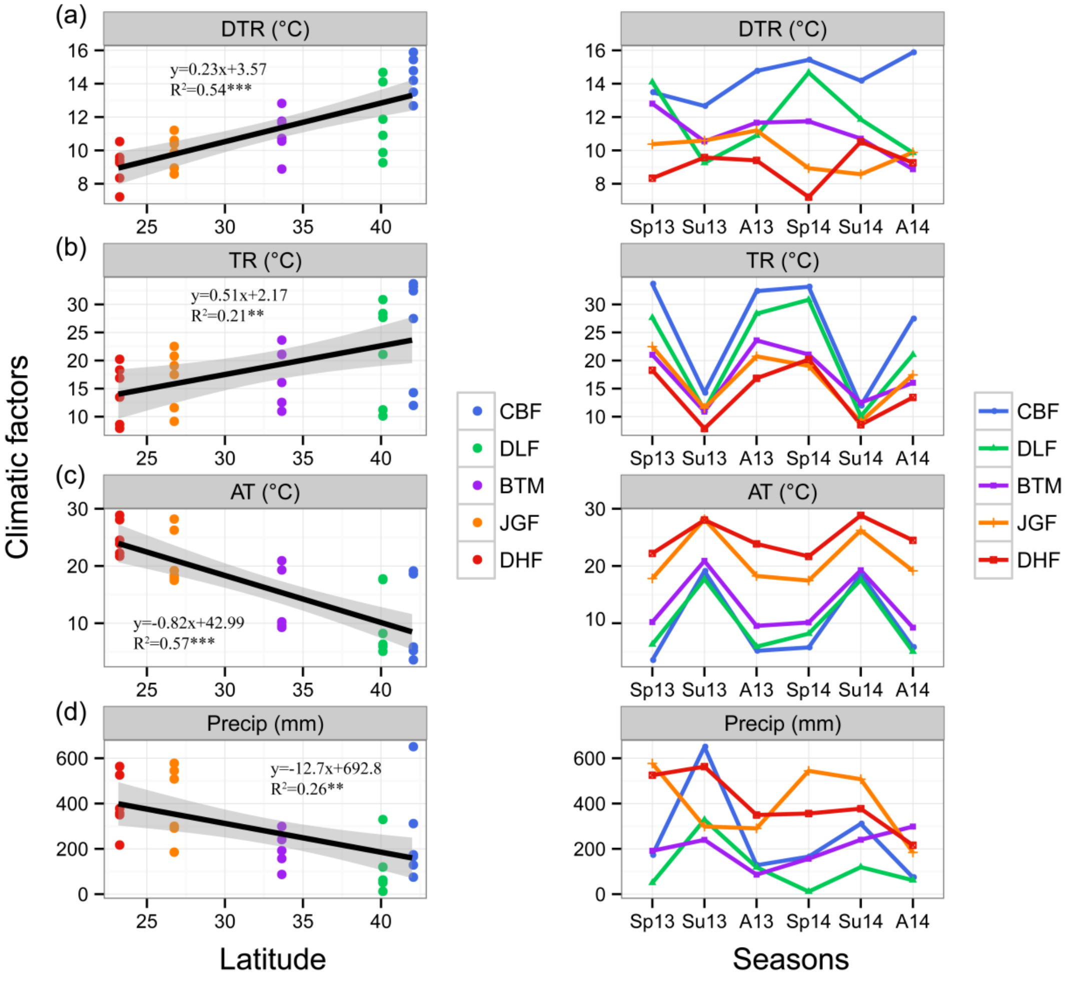

Figure S3. Latitudinal and seasonal changes of climatic factors across latitudinal forest ecosystems. Black lines represent the linear regression fits. (a) DTR: intra-seasonal mean diurnal temperature range. (b) TR: intra-seasonal temperature range. (c) AT: intra-seasonal mean temperature. (d) Precip: intra-seasonal sum of precipitation.

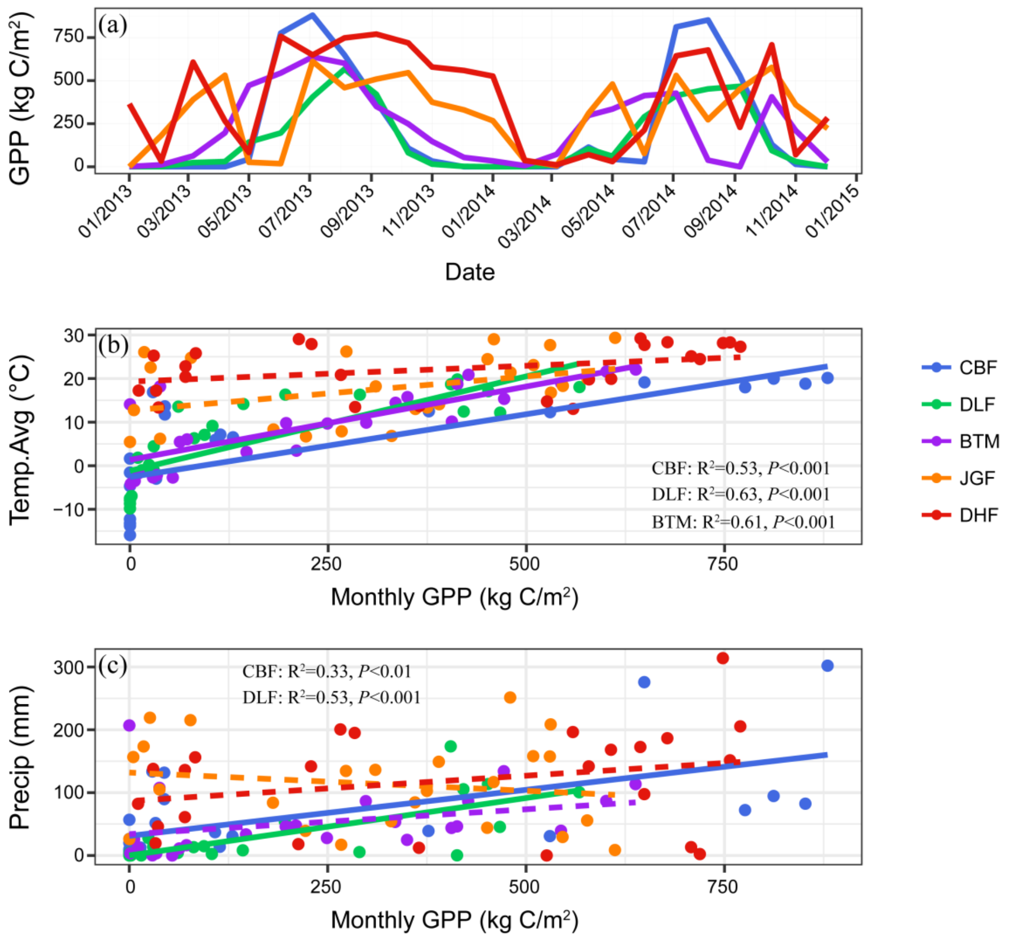

Figure S4. Gross primary productivity (GPP, a) and the relationship with monthly temperature average (Temp.Avg, b) and precipitation (Precip, c). Lines are the linear regression fits. Linear relationships at different latitudinal forests are indicated by color. The solid lines indicate significant relationships (*P*<0.05), while dotted lines indicate insignificant relationships (*P*>0.05).

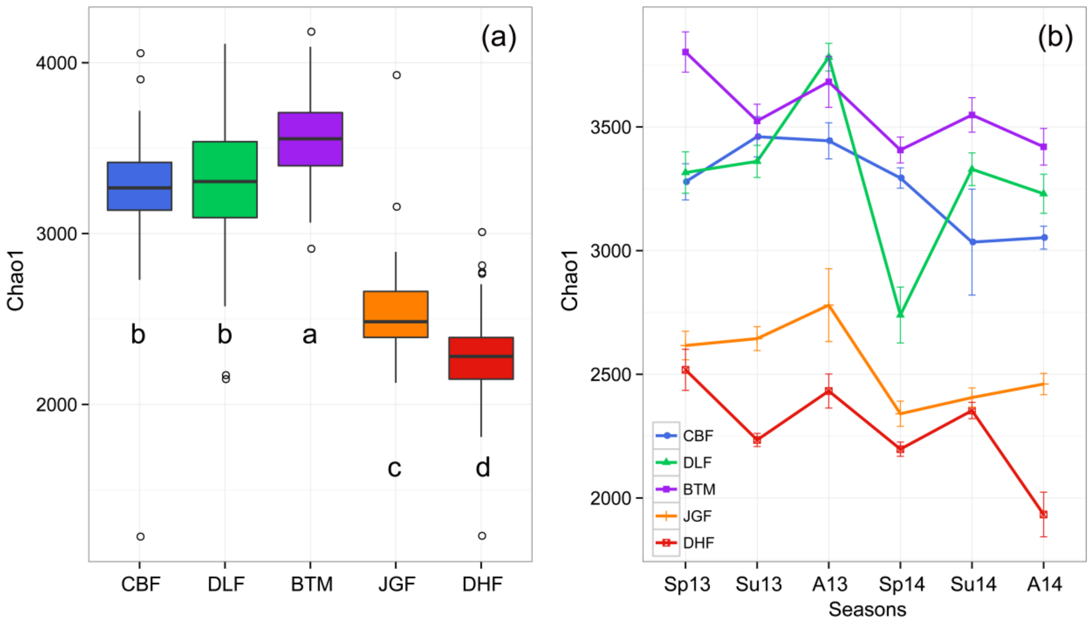

Figure S5. Latitudinal (a) and seasonal (b) changes of OTU richness (Chao1) across latitudinal forest ecosystems. Pairwise comparison was performed between latitudinal samples. Different letters (a, b, c, d) indicate a significant difference ($P<0.05$) by ANOVA analysis. Data on the right figure are means ±SE, n=10.

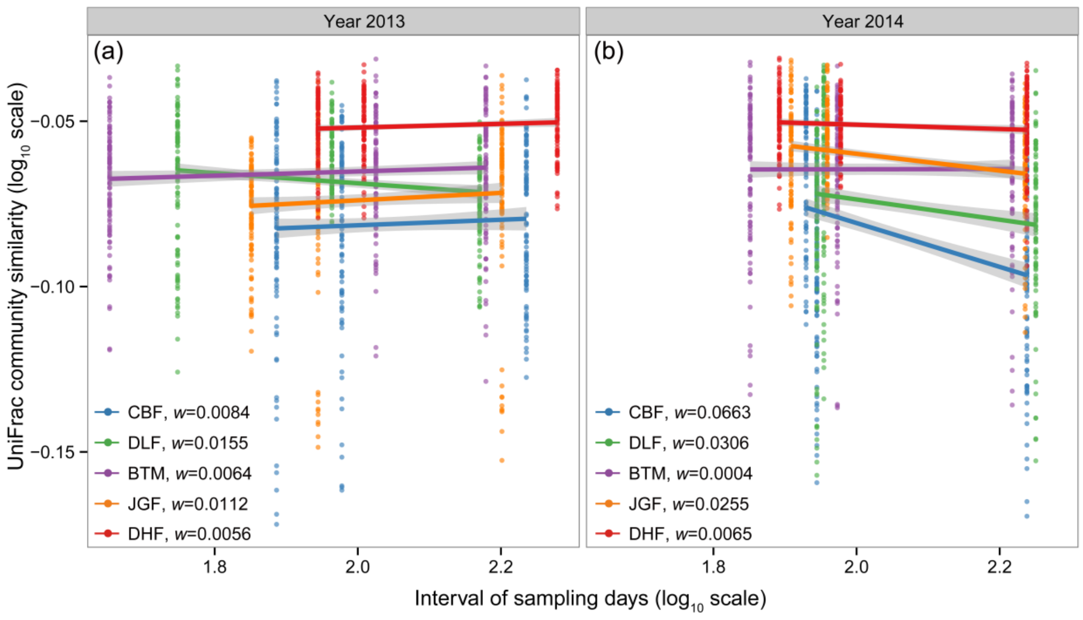

Figure S6. Time-decay for similarity relationship for the microbial community across latitudinal forest ecosystems at Year 2013 (a) and 2014 (b). The turnover rate, *w* (the regression slope), is estimated using a linear regression (log-log space approach) fit between the pairwise average similarity values and intervals of sampling time. The slopes of all lines are significantly different from zero and significantly different for pairwise comparison. Solid lines indicate the relationships are significant (P<0.05) based on linear regression estimated using ordinary least squares. Linear relationships at different latitudinal forests are indicated by color, and the shaded region represents the 95% confidence limits on the regression estimates.

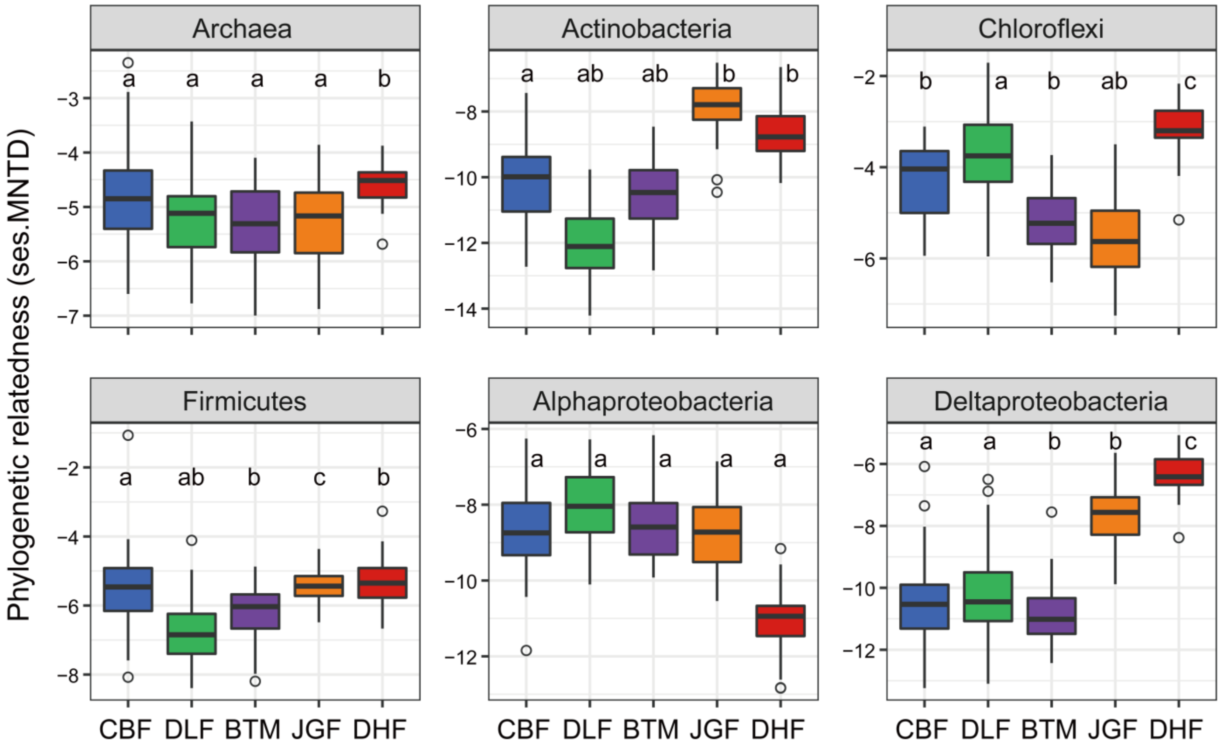

Figure S7. The phylogenetic relatedness [standardized effect sizes of mean nearest taxon distance (ses.MNTD)] for the phylogenetic groups of microbial communities across latitudinal forest ecosystems. The groups whose turnover rates had strong correlations with temperature variation were shown here. Different letters (a, b, c) indicate a significant difference (P<0.05) in the variances of ses.MNTD values between latitudinal forests by F test. The greater variance of ses.MNTD values indicated that species were more phylogenetically dispersed in a community, that is, more dynamic seasonally.

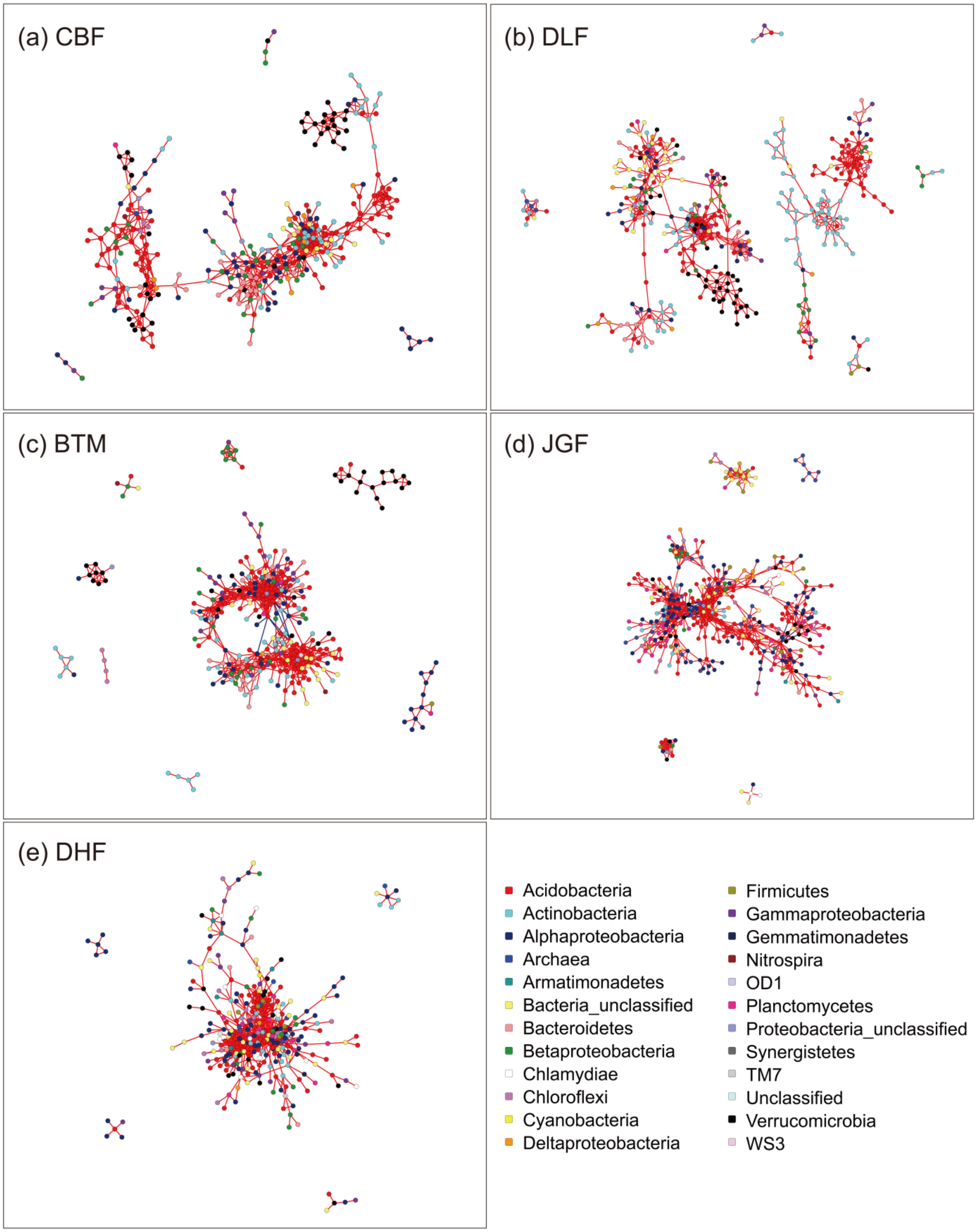

Figure S8. The network graph for the microbial community across latitudinal forest ecosystems: (a) CBF, (b) DLF, (c) BTM, (d) JGF and (e) DHF by the fast greedy modularity optimization method. Each node represents a microbial OTU. Colors of the nodes indicate OTUs affiliated to different major phyla and classes of *Proteobacteria*. Each edge connects two OTUs. A red edge indicates a positive interaction between two individual nodes suggesting a mutualism or cooperation, whereas a blue edge indicates a negative interaction suggesting predation or competition.

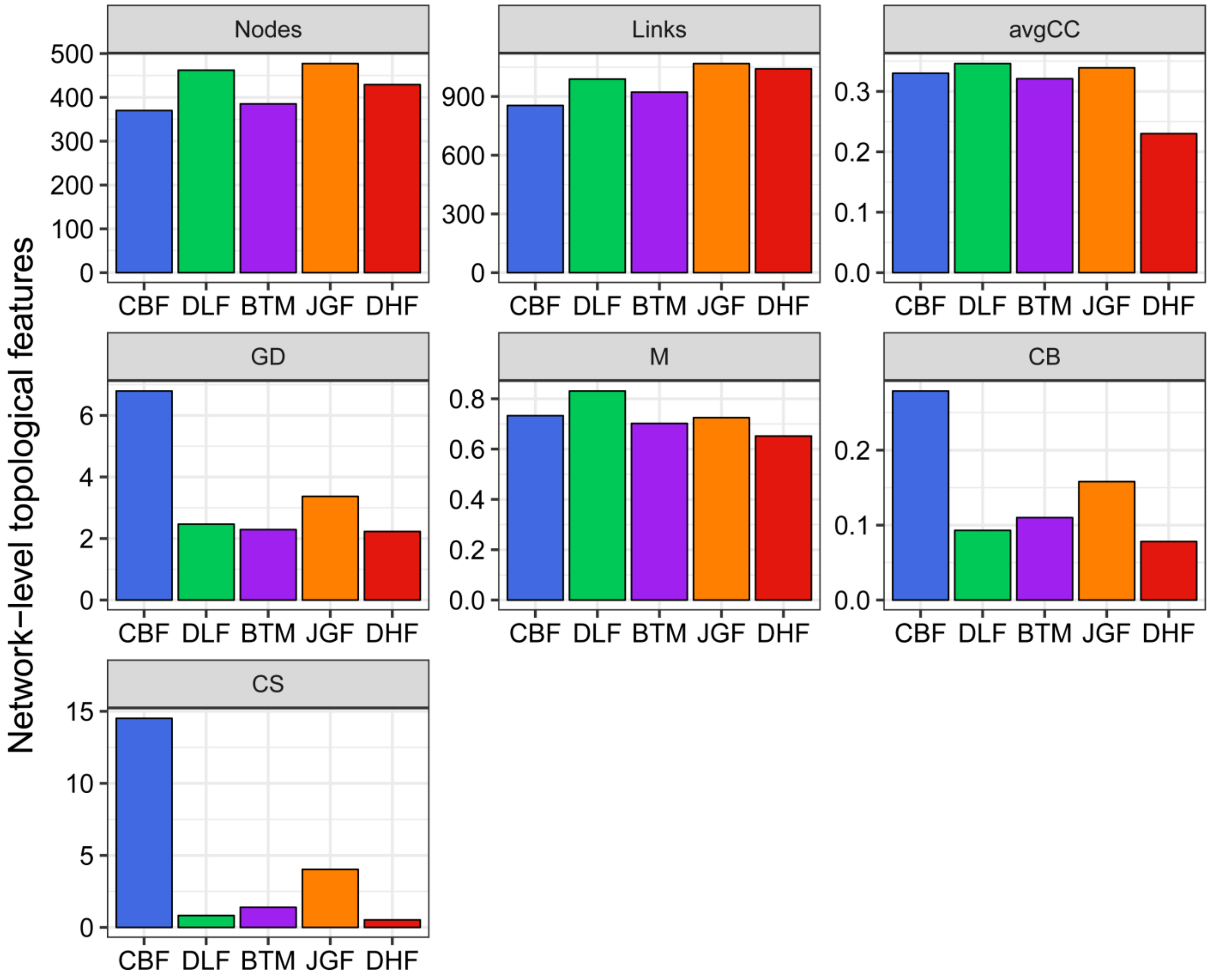

Figure S9. Network-level topological features for the microbial community across latitudinal forest ecosystems. Nodes: node numbers. Links: edge numbers. avgCC: average clustering coefficient. GD: average path distance. M: modularity. CB: centralization of betweenness. CS: centralization of stress.

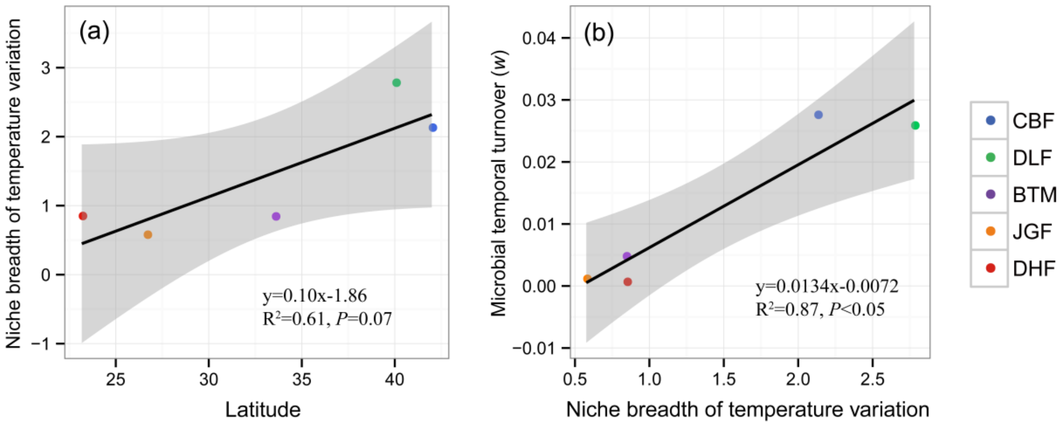

Figure S10. Niche breadth of temperature variation along a latitudinal gradient (a) and the relationship with temporal turnover rates of microbial communities (b). Black lines are the linear regression fits. The shaded region represents the 95% confidence limits on the regression estimates.

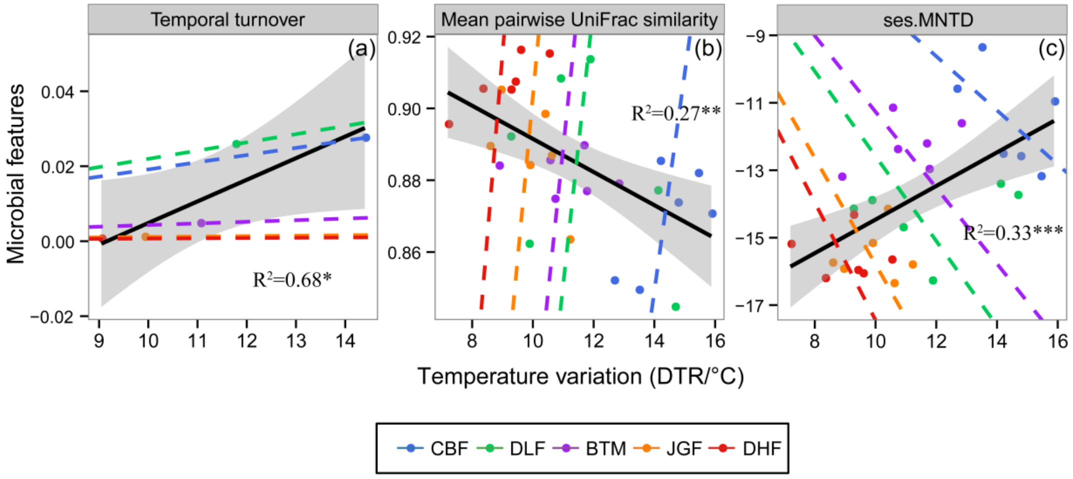

Figure S11. The relationships between microbial community dynamics and temperature variation (DTR). Microbial community dynamics include temporal turnover rate (*w* values, a), mean pairwise UniFrac similarity (b) and phylogenetic relatedness (ses.MNTD, c). The dotted lines indicate average net microbial community dynamics plotted as a function of rates of temperature variation. The figure illustrated that higher latitudes caused greater microbial community dynamics per unit variation of diurnal temperature. Solid black lines are the linear regression fits. The shaded region represents the 95% confidence limits on the regression estimates. Asterisks represent significance level: *** P<0.001, ** P<0.01, * P<0.05. DTR: intra-seasonal mean diurnal temperature range.

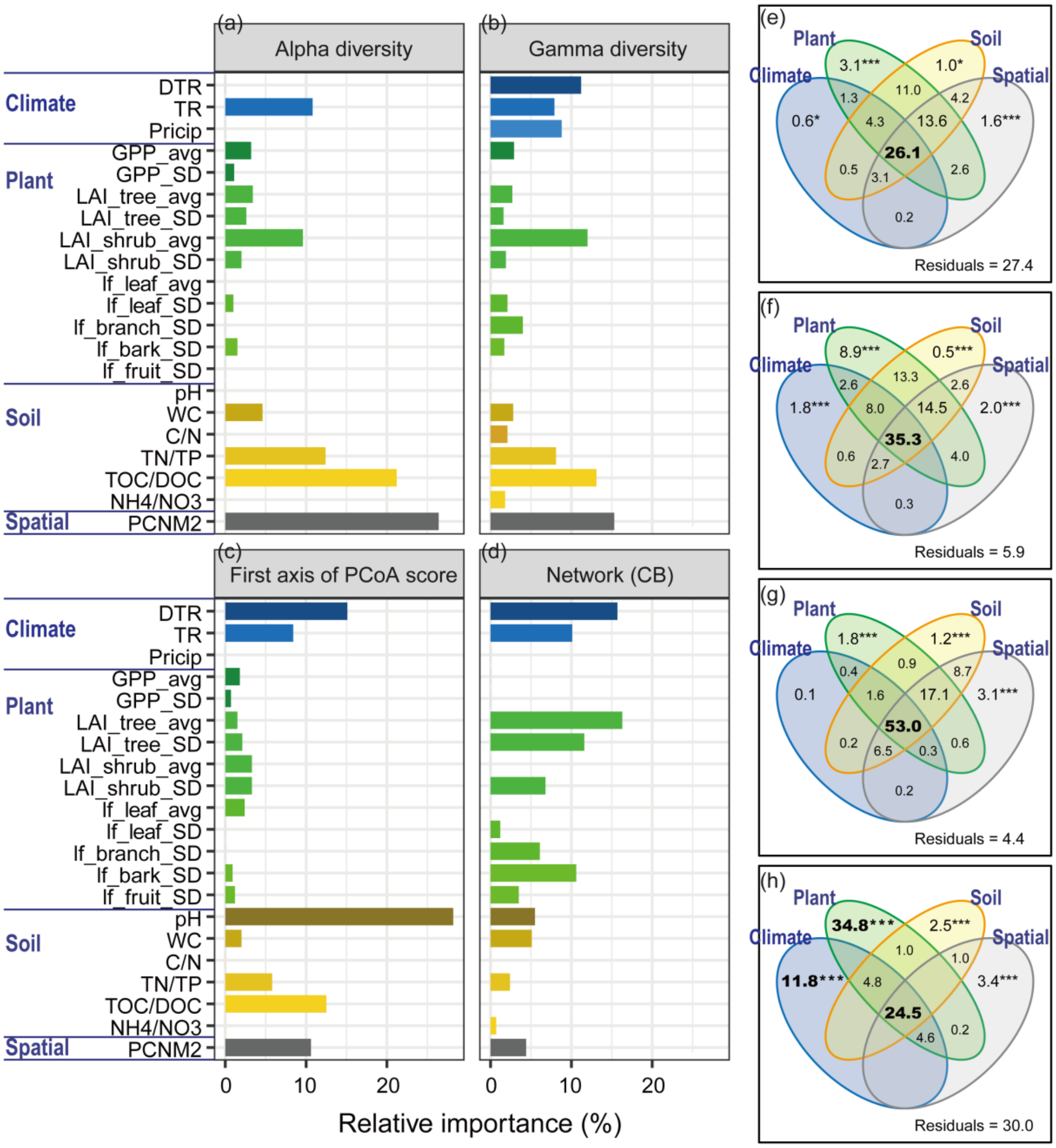

(a)
Alpha diversity
(b)
Gamma diversity
(c)
First axis of PCoA score
(d)
Network (CB)
Climate
Plant
Soil
Spatial
DTR
TR
Pricip
GPP_avg
GPP_SD
LAI_tree_avg
LAI_tree_SD
LAI_shrub_avg
LAI_shrub_SD
lf_leaf_avg
lf_leaf_SD
lf_branch_SD
lf_bark_SD
lf_fruit_SD
pH
WC
C/N
TN/TP
TOC/DOC
NH4/NO3
PCNM2
0
10
20
Relative importance (%)
(e)
Plant
Soil
Climate
Spatial
3.1***
1.0*
1.3
11.0
4.2
0.6*
4.3
13.6
1.6***
26.1
0.5
3.1
2.6
0.2
Residuals = 27.4
(f)
8.9***
0.5***
2.6
13.3
2.6
1.8***
8.0
14.5
2.0***
35.3
0.6
2.7
4.0
0.3
Residuals = 5.9
(g)
1.8***
1.2***
0.4
0.9
8.7
0.1
1.6
17.1
3.1***
53.0
0.2
6.5
0.3
0.6
0.2
Residuals = 4.4
(h)
34.8***
2.5***
1.0
1.0
11.8***
4.8
3.4***
24.5
4.6
0.2
Residuals = 30.0

Figure S12. The relative importance of environmental factors related to the microbial features, identified with a linear model based on multiple ordinary least squares (OLS) regression (a-d) and variation partition analysis (VPA, e-h). Microbial features include alpha-diversity (a and e), gamma-diversity (b and f), community structure (the first axis of PCoA scores, c and g) and network-level topological features (CB; d and h). The values of the relative importance (%) of each variable for each microbial metric in the model are shown as bar plots. The best models were identified using Akaike's information criterion. All of the environmental variables were standardized (mean=0; SD=1). CB: centralization of betweenness. Environmental factors are divided into groups of climate (DTR: intra-seasonal mean diurnal temperature range, TR: intra-seasonal temperature range, and Precip: intra-seasonal sum of precipitation), plant (GPP_avg: intra-seasonal mean of GPP, GPP_SD: intra-seasonal standard deviation of GPP, LAI_tree_avg: intra-seasonal mean of tree leaf area index, LAI_tree_SD: intra-seasonal standard deviation of tree leaf area index, LAI_shrub_avg: intra-seasonal mean of shrub leaf area index, LAI_shrub_SD: intra-seasonal standard deviation of shrub leaf area index, lf_leaf_avg: intra-seasonal mean of leaf litterfall, lf_leaf_SD: intra-seasonal standard deviation of leaf litterfall, lf_branch_SD: intra-seasonal standard deviation of branch litterfall, lf_bark_SD: intra-seasonal standard deviation of bark litterfall, and lf_fruit_SD: intra-seasonal standard deviation of fruit litterfall), soil (pH: soil pH, WC: water content, C/N: the ratio of total organic carbon and nitrogen, TN/TP: the ratio of total nitrogen and phosphorus, TOC/DOC: the ratio of total organic carbon and dissolved organic carbon, and NH4+/NO3-: the ratio of ammonium and nitrate.) and spatial (PCNM2) variables. Asterisks represent significance level: *** $P<0.001$, ** $P<0.01$, * $P<0.05$.

Supplementary Tables

Table S1. Summary of main characteristics of sampling sites along the latitudinal gradient in Eastern China. Abbreviations: MAT, mean annual temperature over two years from 2013 to 2014; MAP, mean annual precipitation over two years from 2013 to 2014; CBF, Changbai Forest; DLF, Dongling Forest; BTM, Baotianman Forest; JGF, Jinggang Forest; and DHF, Dinghu Forest.

Sampling sites	Forest types	Dominant tree species	Soil types	Location	Elevation (m)	MAT (℃)	MAP (mm)
**CBF**	temperate forest	*Pinus koraiensis*	dark brown forest earth	41°59'17''N, 127°56'19''E	760	4.0	769
**DLF**	warm temperate forest	*Liaotungensis*	brown earth	40°02'03''N, 115°27'41''E	1,300	5.1	349
**BTM**	warm temperate to north subtropical forest	*Quercus*	yellow brown earth	33°33'34''N, 111°42'29''E	1,400	9.3	629
**JGF**	middle subtropical forest	*Castanopsis eyrei*, *Pinus massoniana*, *Phyllostachys heterocycla*	mountain yellow earth	26°40'01''N, 114°23'34''E	480	17.7	1,364
**DHF**	southern subtropical forest	*Chinquapin*, *Schima superba*	lateritic red earth	23°10'30''N, 112°33'12''E	470	22.2	1,428

Table S2. Quantitative effects of latitudes and seasons on the variation in the microbial community with adonis based on the weighted UniFrac distance. adonis: permutational multivariate analysis of variance (PERMANOVA) with the adonis function. The R^2 value is the proportion of the community variances constrained by the factor.

	Latitudes		Seasons		Latitudes:Seasons	
	R^2	*P*	R^2	*P*	R^2	*P*
Community structure	0.9081	0.001	0.0027	0.01	0.0077	0.001

Table S3. Temporal turnover rates of microbial communities among different phylogenetic groups.

	**CBF**			**DLF**			**BTM**			**JGF**			**DHF**		
	w	**t**	***P***	***w***	**t**	***P***	***w***	**t**	***P***	***w***	**t**	***P***	***w***	**t**	***P***
**all OTUs**	0.0276	-509.7	<0.001	0.0259	-682.9	<0.001	0.0048	168.2	<0.001	0.0011	-27.8	<0.001	0.0006	-30.9	<0.001
Archaea	0.0372	-411.4	<0.001	0.0160	142.1	<0.001	0.0082	-106.9	<0.001	0.0113	-251.0	<0.001	0.0029	-196.3	<0.001
**Bacterial phyla**															
Acidobacteria	0.0080	-75.8	<0.001	0.0111	-135.7	<0.001	0.0153	202.2	<0.001	0.0094	-188.5	<0.001	0.0054	-250.8	<0.001
Actinobacteria	0.0084	-232.7	<0.001	0.0154	-570.8	<0.001	0.0019	-93.9	<0.001	0.0067	-157.1	<0.001	0.0027	193.3	<0.001
Bacteroidetes	0.0094	-137.8	<0.001	0.0142	-488.5	<0.001	0.0001	-0.2	0.8658	0.0275	290.9	<0.001	0.0076	-175.0	<0.001
Chloroflexi	0.1200	-412.8	<0.001	0.0273	-112.6	<0.001	0.0234	-187.6	<0.001	0.0177	-356.8	<0.001	0.0080	-307.3	<0.001
Firmicutes	0.1136	-515.9	<0.001	0.0124	-99.8	<0.001	0.0012	18.3	<0.001	0.0106	143.8	<0.001	0.0092	136.2	<0.001
Planctomycetes	0.0216	-380.0	<0.001	0.0335	-606.8	<0.001	0.0112	356.1	<0.001	0.0239	-261.8	<0.001	0.0116	252.9	<0.001
Verrucomicrobia	0.0006	-16.3	<0.001	0.0016	97.0	<0.001	0.0011	-103.1	<0.001	0.0030	-56.1	<0.001	0.0049	191.8	<0.001
***Proteobacterial* classes**															
Alphaproteobacteria	0.0132	630.2	<0.001	0.0041	-196.4	<0.001	0.0071	359.2	<0.001	0.0032	156.9	<0.001	0.0039	-328.8	<0.001
Betaproteobacteria	0.0096	-379.2	<0.001	0.0043	-135.0	<0.001	0.0017	85.4	<0.001	0.0050	-141.5	<0.001	0.0116	-406.1	<0.001
Gammaproteobacteria	0.0008	-20.9	<0.001	0.0235	-520.3	<0.001	0.0047	234.7	<0.001	0.0030	-98.7	<0.001	0.0113	-452.2	<0.001
Deltaproteobacteria	0.0210	-554.4	<0.001	0.0083	-276.6	<0.001	0.0015	-84.7	<0.001	0.0028	85.3	<0.001	0.0032	-100.3	<0.001

The turnover rates (*w* values) shown were determined using the time-decay approach. t and *P*-values are from one-sample *t*-test on bootstrapping (1000 times) for testing significance of *w* values. Forests along a latitudinal gradient from north to south include Changbai Forest (CBF), Dongling Forest (DLF), Baotianman Forest (BTM), Jinggang Forest (JGF) and Dinghu Forest (DHF).

Table S4. Topological properties of the microbial communities' phylogenetic molecular ecological networks (MENs) across latitudinal forest ecosystems and their associated random MENs. [a]Significant difference between experimental network and random network by the *t*-test (P<0.001).

	Empirical networks									Random networks		
	Similarity threshold (st)	Node numbers (Nodes)	Edge numbers (Links)			R^2 of power law	Average path distance (GD)	Average clustering coefficient (avgCC)	Modularity (M)	Average path distance (GD)	Average clustering coefficient (avgCC)	Modularity (M)
				positive (%)	negative (%)							
**CBF**	0.73	370	854	100	0	0.883	6.794	0.330	0.733	3.446 ± 0.109	0.035 ± 0.006	0.440 ± 0.006
**DLF**	0.72	462	989	100	0	0.930	2.467	0.346	0.831	3.624 ± 0.113	0.025 ± 0.005	0.472 ± 0.005
**BTM**	0.73	385	922	98.6	1.4	0.902	2.289	0.321	0.701	3.411 ± 0.110	0.035 ± 0.005	0.429 ± 0.006
**JGF**	0.72	477	1068	96.9	3.1	0.912	3.369	0.339	0.725	3.589 ± 0.114	0.022 ± 0.004	0.460 ± 0.006
**DHF**	0.73	429	1041	99.8	0.2	0.841	2.229	0.230	0.652	3.054 ± 0.121	0.061 ± 0.007	0.401 ± 0.005

Table S5. Temporal turnover rates of microbial communities among different phylogenetic groups at the genus level, and their relationships with climatic factors. The turnover rates (*w* values) shown were determined using the time-decay approach. Significant *w* (P<0.001) were shown here. Pearson correlation coefficients (r) between temporal turnover rate for each phylogenetic group of microbial communities and climatic factors across latitudinal forest ecosystems. The significant (P<0.05) correlation coefficients were indicated in bold. DTR: intra-seasonal mean diurnal temperature range. TR: intra-seasonal temperature range. AT: intra-seasonal mean temperature. Precip: intra-seasonal sum of precipitation.

**Phylogenetic groups**	**Genus**	**DTR**		**TR**		**AT**		**Precip**	
		r	***P***	***r***	***P***	***r***	***P***	***r***	***P***
**Archaea**	*Fervidicoccus*	0.443	0.455	0.308	0.614	-0.074	0.906	0.353	0.560
	Unclassified	0.858	0.063	**0.891**	**0.042**	-0.642	0.243	-0.408	0.495
Chloroflexi	*Ktedonobacter*	0.250	0.686	0.337	0.579	-0.643	0.242	**-0.909**	**0.032**
	Unclassified	0.015	0.981	0.015	0.981	0.375	0.534	0.730	0.161
Alphaproteobacteria	*Acidisoma*	0.692	0.195	0.629	0.256	-0.247	0.689	0.183	0.769
	Anderseniella	0.922	0.252	0.753	0.458	-0.434	0.714	0.879	0.316
	Bradyrhizobium	-0.824	0.086	**-0.888**	**0.044**	**0.894**	**0.041**	0.707	0.182
	Devosia	-0.447	0.705	-0.141	0.910	-0.259	0.833	-0.977	0.137
	Dongia	-0.719	0.171	-0.561	0.326	0.529	0.360	0.207	0.738
	Hyphomicrobium	-0.617	0.383	-0.532	0.468	0.922	0.078	0.856	0.144
	Mesorhizobium	0.788	0.422	0.553	0.627	-0.182	0.883	0.974	0.147
	Methylocystis	-0.033	0.958	-0.083	0.894	-0.409	0.494	-0.643	0.242
	Pedomicrobium	0.115	0.885	0.312	0.688	-0.530	0.470	-0.759	0.241
	Phenylobacterium	0.387	0.520	0.565	0.321	-0.409	0.494	-0.419	0.483
	Pseudolabrys	-0.589	0.296	-0.601	0.284	0.715	0.175	0.520	0.370
	Rhizobium	**0.998**	**0.042**	0.925	0.248	-0.703	0.504	0.678	0.526
	Rhizomicrobium	**-0.887**	**0.045**	**-0.945**	**0.016**	0.729	0.162	0.408	0.496
	Rhodomicrobium	-0.573	0.312	-0.445	0.452	0.481	0.412	0.154	0.804
	Rhodoplanes	-0.266	0.665	-0.098	0.876	0.094	0.881	-0.212	0.733
	Skermanella	-0.317	0.683	-0.201	0.799	0.398	0.602	0.589	0.411
	Sphingomonas	0.984	0.115	0.990	0.091	-0.855	0.347	0.478	0.683
	Sphingosinicella	0.899	0.288	0.991	0.083	-0.963	0.173	0.223	0.857
	Stella	0.471	0.423	0.446	0.452	-0.149	0.811	0.003	0.996
	Unclassified	0.484	0.409	0.302	0.622	-0.452	0.444	-0.258	0.675
Deltaproteobacteria	*Anaeromyxobacter*	0.189	0.761	0.315	0.605	-0.573	0.312	-0.837	0.077
	Bdellovibrio	0.186	0.765	0.248	0.688	0.139	0.824	0.488	0.404
	Byssovorax	-0.351	0.649	-0.149	0.851	0.577	0.423	0.526	0.474
	Chondromyces	-0.639	0.246	-0.656	0.229	0.790	0.112	0.619	0.265
	Cystobacter	-0.984	0.116	-0.990	0.090	0.856	0.346	-0.476	0.684
	Haliangium	-0.627	0.258	-0.646	0.239	0.759	0.137	0.571	0.314

	Kofleria	-0.854	0.348	-0.758	0.452	**0.999**	**0.032**	0.883	0.311
	Phaselicystis	-0.283	0.818	-0.572	0.612	0.848	0.356	0.570	0.614
	Unclassified	-0.318	0.602	-0.179	0.773	0.187	0.763	-0.130	0.834
	Vampirovibrio	0.255	0.679	0.376	0.533	-0.566	0.320	-0.816	0.092

Table S6. Relationships between microbial features and potential explanatory variables that were modeled using multiple ordinary least squares (OLS) regression. Microbial features include temporal turnover rate, mean pairwise UniFrac similarity, phylogenetic relatedness (ses.MNTD) and network-level topological features. The best models were identified using Akaike's information criterion (AIC). GD: average path distance. avgCC: average clustering coefficient. CB: centralization of betweenness. CS: centralization of stress. DTR: intra-seasonal mean diurnal temperature range. TR: intra-seasonal temperature range. Precip: intra-seasonal sum of precipitation. GPP_avg: intra-seasonal mean of GPP. GPP_SD: intra-seasonal standard deviation of GPP. LAI_tree_avg: intra-seasonal mean of tree leaf area index. LAI_tree_SD: intra-seasonal standard deviation of tree leaf area index. LAI_shrub_avg: intra-seasonal mean of shrub leaf area index. LAI_shrub_SD: intra-seasonal standard deviation of shrub leaf area index. lf_leaf_avg: intra-seasonal mean of leaf litterfall. lf_leaf_SD: intra-seasonal standard deviation of leaf litterfall. lf_branch_SD: intra-seasonal standard deviation of branch litterfall. lf_bark_SD: intra-seasonal standard deviation of bark litterfall. lf_fruit_SD: intra-seasonal standard deviation of fruit litterfall. pH: soil pH. WC: water content. C/N: the ratio of total organic carbon and nitrogen. TN/TP: the ratio of total nitrogen and phosphorus. TOC/DOC: the ratio of total organic carbon and dissolved organic carbon.

	AIC	R^2	**Explanatory variables and β-weights**							
Temporal turnover	-392.7	0.744	DTR<br>[a]0.35***	Precip<br>-0.42***	GPP_SD<br>0.31***	lf_branch_SD<br>0.28***	lf_bark_SD<br>-0.37***	LAI_tree_avg<br>-0.12**	PCNM2<br>-0.49***	C/N<br>0.08*
Mean pairwise similarity	-338.0	0.694	DTR<br>-0.17*	Precip<br>0.26***	GPP_avg<br>-0.35***	LAI_shrub_avg<br>1.10***	LAI_tree_avg<br>-0.54***	LAI_shrub_SD<br>-0.70***	lf_fruit_SD<br>0.48***	PCNM2<br>0.34***
ses.MNTD	-203.3	0.510	DTR<br>0.22***	TR<br>0.18**	LAI_tree_avg<br>0.40***	lf_bark_SD<br>0.21***	LAI_tree_SD<br>0.13**	LAI_shrub_avg<br>-0.15*	C/N<br>-0.22***	TN/TP<br>-0.18**
Alpha diversity	-372.7	0.724	TR<br>0.19**	GPP_avg<br>0.37***	LAI_shrub_avg<br>-0.34***	lf_bark_SD<br>0.17***	LAI_shrub_SD<br>0.28***	LAI_tree_avg<br>0.19***	PCNM2<br>0.66***	TOC/DOC<br>0.21***
Gamma diversity	-734.1	0.918	DTR<br>0.18***	GPP_avg<br>0.38***	LAI_shrub_avg<br>-0.59***	LAI_tree_avg<br>0.27***	lf_bark_SD<br>0.19***	LAI_shrub_SD<br>0.31***	lf_leaf_SD<br>0.17***	PCNM2<br>0.55***
Community structure	-799.0	0.934	DTR<br>0.18***	GPP_avg<br>0.21***	GPP_SD<br>0.10***	LAI_shrub_SD<br>0.35***	LAI_tree_avg<br>0.17***	LAI_tree_SD<br>0.19***	PCNM2<br>0.32***	pH<br>0.46***
GD	-245.3	0.577	DTR<br>0.45***	GPP_SD<br>-0.13***	lf_branch_SD<br>-0.44***	LAI_tree_SD<br>-0.50***	LAI_tree_avg<br>-0.35***	LAI_shrub_avg<br>-0.38***	lf_leaf_avg<br>0.31***	PCNM2<br>-0.28***
avgCC	-261.7	0.602	DTR<br>0.50***	Precip<br>-0.31***	lf_branch_SD<br>-0.19***	LAI_tree_avg<br>0.20**	lf_bark_SD<br>-0.15**	LAI_shrub_avg<br>0.18*	PCNM2<br>-0.56***	WC<br>-0.21**
CS	-222.5	0.545	DTR<br>0.24**	Precip<br>-0.41***	GPP_SD<br>0.15**	lf_bark_SD<br>-0.25***	LAI_shrub_SD<br>0.30***	lf_fruit_SD<br>-0.23***	PCNM2<br>-0.40***	C/N<br>0.24***
CB	-281.0	0.627	DTR<br>0.30***	DTR<br>0.26***	LAI_tree_avg<br>0.53***	lf_fruit_SD<br>-0.43***	LAI_shrub_SD<br>0.33***	LAI_tree_SD<br>0.25***	PCNM2<br>-0.39***	WC<br>-0.29***

[a]Standardized partial regression coefficients. * $P<0.05$, ** $P<0.01$, *** $P<0.001$.
